# Unexpected complexity of the ammonia monooxygenase in archaea

**DOI:** 10.1038/s41396-023-01367-3

**Published:** 2023-01-31

**Authors:** Logan H. Hodgskiss, Michael Melcher, Melina Kerou, Weiqiang Chen, Rafael I. Ponce-Toledo, Savvas N. Savvides, Stefanie Wienkoop, Markus Hartl, Christa Schleper

**Affiliations:** 1 Archaea Biology and Ecogenomics Unit, Department of Functional and Evolutionary Ecology, University of Vienna, Vienna, Austria; 2 Mass Spectrometry Facility, Max Perutz Labs, Vienna BioCenter (VBC), Vienna, Austria; 3 VIB Center for Inflammation Center and Department of Biochemistry & Microbiology, Ghent University, Ghent, Belgium; 4 Molecular Systems Biology Unit, Department of Functional and Evolutionary Ecology, University of Vienna, Vienna, Austria; 5 Department of Biochemistry and Cell Biology, Max Perutz Labs, University of Vienna, Vienna, Austria

**Keywords:** Archaeal physiology, Soil microbiology, Metabolism, Environmental sciences, Proteomics

## Abstract

Ammonia oxidation, as the first step of nitrification, constitutes a critical process in the global nitrogen cycle. However, fundamental knowledge of its key enzyme, the copper-dependent ammonia monooxygenase, is lacking, in particular for the environmentally abundant ammonia-oxidizing archaea (AOA). Here the structure of the enzyme is investigated by blue-native gel electrophoresis and proteomics from native membrane complexes of two AOA. Besides the known AmoABC subunits and the earlier predicted AmoX, two new protein subunits, AmoY and AmoZ, were identified. They are unique to AOA, highly conserved and co-regulated, and their genes are linked to other AMO subunit genes in streamlined AOA genomes. Modeling and in-gel cross-link approaches support an overall protomer structure similar to the distantly related bacterial particulate methane monooxygenase but also reveals clear differences in extracellular domains of the enzyme. These data open avenues for further structure-function studies of this ecologically important nitrification complex.

## Introduction

Nitrification, the conversion of ammonium to nitrate, is a crucial step in the global nitrogen cycle solely performed by microorganisms. The process has attracted particular attention due to its agricultural and environmental relevance. The first and rate limiting [1] step of nitrification is the oxidation of ammonia via the integral membrane protein complex ammonia monooxygenase (AMO) [2, 3]. While ammonia-oxidizing bacteria (AOB) were first discovered over 125 years ago [4] and have been extensively studied, this biological process was also detected in the archaeal domain in the last 20 years [5–7]. Ammonia-oxidizing archaea (AOA) have gained broad attention as they are widespread in nature and are more abundant than their bacterial counterparts in most terrestrial and marine environments, indicating important roles in nitrogen cycling [8–14]. Their central nitrogen and carbon metabolism, however, is distinct from that of AOB [15–18]. In particular, subunits of the AMO complex show only about 40% identity to those of bacteria [19] and archaeal proteins catalyzing the second step in ammonia oxidation, i.e. the conversion of hydroxylamine to nitrite, are still unknown [19–21]. These differences imply important functional differentiation in their environmental roles that have yet to be unraveled.

Due to the difficulty of growing nitrifying organisms and the inherent problems with isolating membrane proteins, no structural studies have been successfully carried out for any AMO complex, bacterial or archaeal. This holds true for most of the diverse enzymes of the CuMMO (copper-dependent membrane monooxygenase) protein family, with a few notable exceptions. Crystal structures [22–26] and cryo-EM structures [27, 28] of particulate methane monooxygenase (pMMO) from five methanotrophs have consistently confirmed a three-polypeptide protomer (subunits-A, -B and -C) arranged in a trimer of α_3_β_3_γ_3_ configuration with at least two conserved metal sites in each protomer. Even so, the elucidation of the active site has remained ambiguous. It was first proposed to reside in the PmoB subunit of pMMO [29]. More recently, a cryo-EM analysis supports the active site primarily being coordinated by PmoA [27], while differing amino acid conservation in Verrucomicrobia [30], a recent spectroscopic analysis [31], and mutagenesis of a hydrocarbon monooxygenase [32], suggest its localization in the PmoC subunit.

Although no AMO structure has been determined experimentally, homology modeling for the AMO of the bacterium *Nitrosomonas europaea* using pMMO as a template supported a homotrimeric structure as well as conservation of the Cu_B_ and Cu_C_ copper sites [33]. The archaeal AMO complex is the most distantly related of all CuMMO proteins [34, 35] and very little is known so far about its structure or function. Based on comparative metagenomics alone, it has been suggested that an additional subunit might be present in the complex, termed AmoX [15, 36].

To gain insights into the overall architecture of the archaeal AMO complex, membrane protein fractions from the well characterized soil AOA, *Nitrososphaera viennensis*, were analyzed biochemically using native gel electrophoresis, mass spectrometry, and chemical cross-linking. Beside the three known AmoABC proteins, three additional potential subunits were identified and one of the six predicted AmoC proteins in *N. viennensis* was recognized as the primary homolog in the protein complex. In addition, the overall subunit composition of the AMO complex was confirmed in the distantly related thermophilic AOA *Nitrosocaldus cavascurensis*.

## Materials and methods

### Reactor growth

*Nitrososphaera viennensis* was grown as a continuous culture in 2 L bioreactors (Eppendorf) filled with 1.5 L of freshwater medium (FWM) [37, 38] with modified trace element solution [5], 7.5 µM FeNaEDTA, 2 mM NH_4_Cl, and 1 mM pyruvate at 42 °C and pH 7.5. Carbonate was supplied by gassing the reactors with a 98% air 2% CO_2_ mixture. The applied dilution rates ranged from 0.035 to 0.07 h^−1^.

*Nitrosocaldus cavascurensis* was grown as a batch culture in the same reactors, volume, and medium as described for *N. viennensis*, but at 68 °C with 1 mM NH_4_Cl, 1 mM pyruvate, and pH 7.0. Carbonate was also supplied by gassing, but with a mixture of air/ N_2_/ CO_2_ to achieve a 10% O_2_ and 2% CO_2_ mixture. To increase the biomass, NH_4_Cl was added stepwise with syringes via a septum to increase the final NO_2_^−^ concentration to approximately 2.5 mM before harvesting the cultures.

Harvested biomass was concentrated in three centrifugation steps. First with a continuous centrifuge (CEPA model LE) operating at maximum speed. Biomass from the continuous centrifuge was then suspended in 400 mL volumes and concentrated using a Sorvall Lynx 4000 with an F12–6 × 500 rotor for 30 min at 4 °C and 16,000 × *g*. Finally, biomass was resuspended in small volumes and aliquoted to 1.5 mL Eppendorf tubes and concentrated to a final pellet for 30 min at 4 °C and 16,000 × *g* using a bench-top centrifuge. Pellets were frozen at −70 °C until further analysis.

### Sample and data processing

Detailed information for bioinformatic analysis, membrane protein extraction, BN-PAGE methods, Tricine-SDS-PAGE methods, mass spectrometry preparation, cross-linking, data analysis, and AlphaFold multimer predictions can be found in Supplementary Materials and Methods.

Briefly, cells were lysed and membrane fractions were isolated using ultracentrifugation (Beckman Coulter Ultracentrifuge; SW 41 Ti Swinging-Bucket Rotor, k_max_ = 124; 200,000 × *g*) for 90 min at 4 °C using 13.2 mL thinwall polypropylene tubes with a level of deceleration set to 7. Membrane proteins were extracted using n-dodecyl-β-D-maltoside (DDM; Invitrogen BN2005) and loaded on a 3–12% pre-cast BN-PAGE gel (Invitrogen BN1001). Selected bands were cut out and analyzed via mass spectrometry for protein identification. Procedures for protein extraction and running a BN-PAGE gel were based on previous studies [39, 40] and the NativePAGE Novex Bis-Tris Gel System manual from Life Technologies (MAN0000557). Study design and analysis for membrane extraction and BN-PAGE was guided by previous studies [41, 42] Cross-linking methods were based on protocols from Hevler et al. (2021) [43].

The mass spectrometry proteomics data have been deposited to the ProteomeXchange Consortium via the PRIDE [44] partner repository with the dataset identifiers PXD035349, PXD034632, and PXD034475 for BN-PAGE of *N. viennensis*, BN-PAGE of *N. cavascurensis*, and cross-linked samples, respectively. Relevant scripts for analysis can be found in the GitHub repository https://github.com/hodgskiss/Archaeal_AMO.

## Results

### Complexome analysis of native membrane complexes displays the AMO composition of Nitrososphaera viennensis

**Fig. 1:**
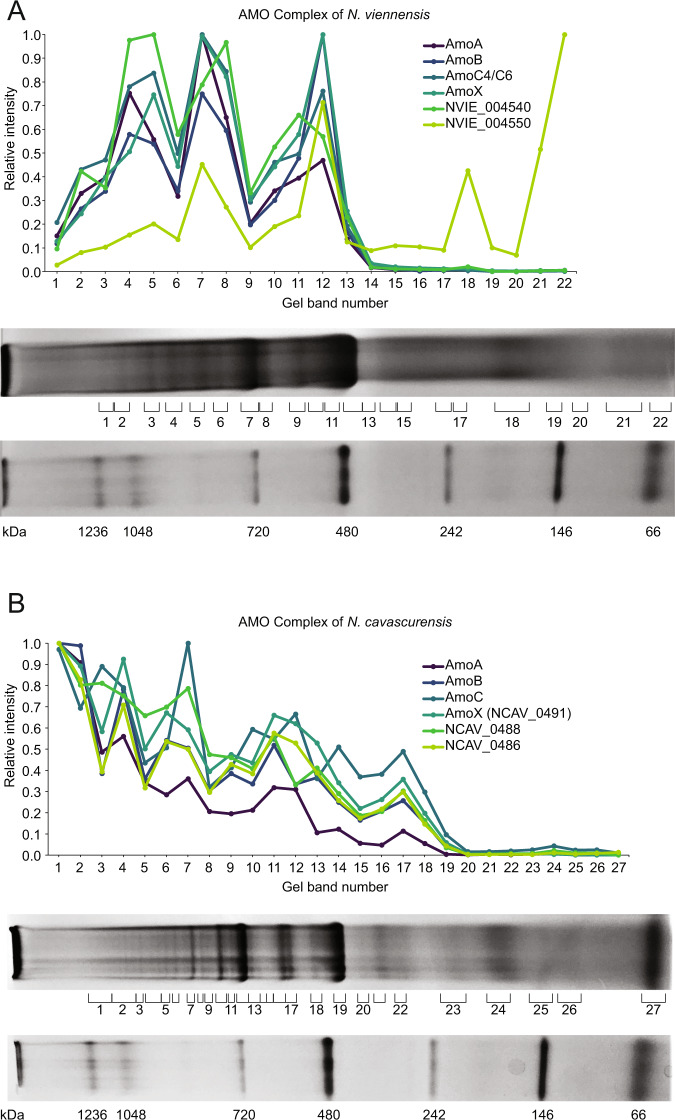
Relative intensity patterns of AMO subunits in BN-PAGE gels. Relative abundance of iBAQ normalized intensities of known and putative AMO subunits. iBAQ intensities for each protein are normalized to the highest detected intensity of that protein to create a relative abundance profile for each protein. **A** Patterns of AMO intensity in *N. viennensis*. **B** Patterns of AMO intensity in *N. cavascurensis*. Bands selected to be cut and analyzed via mass spectroscopy are indicated by numbered brackets from left (top of gel) to right (bottom of gel) and correspond to numbers on the *x*-axis of respective plots. Ladders for each gel are represented at the bottom of the respective panels.

**Fig. 2:**
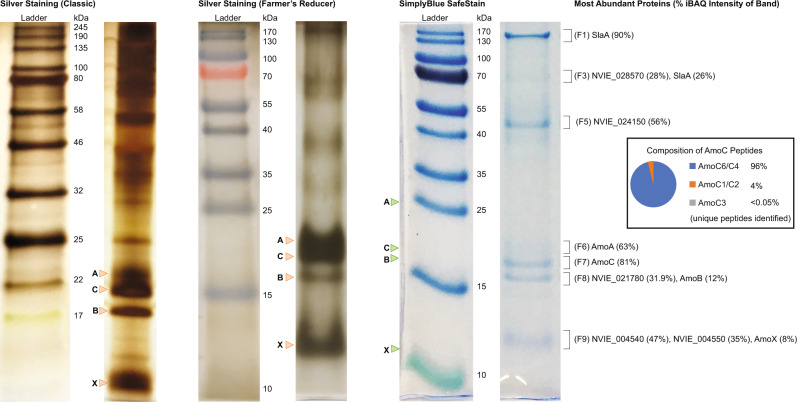
Tricine-SDS-PAGE gels of AMO cut-outs. Tricine-SDS-PAGE of AMO bands from BN-PAGE gels. Comparison of three different staining methods for Tricine-SDS-PAGE gels with size markers on left side. Bands cut for analysis from a gel stained with SimplyBlue SafeStain and digested using trypsin are indicated by brackets. Percentages represent the percentage of iBAQ normalized protein intensities for each individual band. Band identifiers are indicated in parentheses. Green arrows marked A, B, C, and X represent expected heights of bands for AMO subunits AmoA, AmoB, AmoC, and AmoX, respectively. Orange arrows marked A, B, C, and X represent equivalent bands of AmoA, AmoB, AmoC, and AmoX, respectively, from silver-stained gels. A pie chart from the band with the highest amount of AmoC shows the percentage of AmoC bands coming from distinguishable AmoC homologs.

*Nitrososphaera viennensis* was grown in continuous culture for several weeks under optimal growth conditions in order to obtain enough biomass for biochemical analyses (Melcher et al. [45]). Between 800–2000 µg of membrane proteins were obtained from 450–550 mg of biomass per preparation, of which approximately 40–50 µg were loaded per lane on blue-native PAGE gels [39]. After optimization of conditions, 22 bands were cut out and subjected to mass spectrometry (see Supplementary Materials and Methods; Fig. S1A). AMO subunits (AmoA, AmoB, and AmoC) were among the most abundant proteins (22% of iBAQ normalized intensity) detected overall in these membrane fractions. The relative intensity profiles of AmoA, AmoB, and AmoC showed three distinct peaks corresponding to bands 4, 7, and 12, with the most prominent peak occurring at band 7 (Fig. 1A). The subunits AmoA, AmoB, and AmoC made up 10%, 5%, and 14%, respectively, of the total protein found in band 7 based on iBAQ normalized intensities. AmoX was also present in band 7 representing 10%. The most intense signals for the AmoC subunit were represented by two of the six AmoC homologs, AmoC6 and AmoC4. These two homologs could not be distinguished based on the peptides identified in the BN-PAGE gel. In denaturing Tricine-SDS-PAGE of cutouts from band 7, all known components of the AMO complex were visualized and confirmed by proteomics (Fig. 2). In addition, this allowed for the identification of unique peptides of the AmoC6 subunit (see Supplementary Discussion).

To identify additional proteins that might be part of the archaeal AMO complex, a correlation analysis was conducted to find candidates with a similar migration pattern as all three primary AMO subunits AmoA, AmoB, and AmoC4/C6. Patterns of the 50% most abundant proteins were compared to each other using a Kendall correlation to determine the likelihood of dependence between various proteins with a focus on proteins correlated with known AMO subunits. Additional criteria were (i) their presence in fully sequenced AOA, and (ii) their absence in species that do not oxidize ammonia [46]. The two proteins that initially met these criteria were the putative AMO subunit AmoX and a hypothetical protein, NVIE_004540 (Table 1). The migration patterns for these proteins can be seen in Fig. 1A. While this unbiased selection process produced additional AMO candidates, further analysis was needed to verify the presence of these newly identified and other potential subunits.

**Table 1:** Correlations of proteins with occurrence of AmoA,B, and C in (A) *N. viennensis* and (B) *N. cavascurensis* BN-PAGE gels.

Gene	Protein^a^	Conserved in Extant AOA^b^	Exclusive to AOA^b^	Correlations^c^		
				AmoA	AmoB	AmoC6
**A**						
**AMO Correlation Results for** ***N. viennensis***						
NVIE_016740	surface associated S-layer protein		X	X	X	X
*nuoJ*	Complex I	X		X	X	X
*nuoM*	Complex I	X		X	X	X
*coxB*	Complex IV			X	X	X
*coxA1*	Complex IV			X		
NVIE_013530	protein of unknown function		X	X	X	X
NVIE_027260	conserved protein of unknown function		X	X	X	X
NVIE_017130	protein of unknown function DUF373	X		X		X
*amoX*	potential AMO subunit	X	X	X	X	X
NVIE_004540	hypothetical protein	X	X	X	X	X
**B**						
**AMO Correlation Results for** ***N. cavascurensis***						
*coxA*	Cytochrome c oxidase polypeptide 1			X	X	
NCAV_1739	Uncharacterized protein		X	X	X	
*coxB*	Putative heme-copper oxidase subunit II			X	X	X
NCAV_0011	ABC-1 domain-containing protein			X	X	
*amt*	Ammonium transporter	X		X	X	
*petB*	Putative cytochrome b/b6			X		
NCAV_1587	Putative heme/copper-type cytochrome/quinol oxidase, subunit	X	X	X		
NCAV_0486	Uncharacterized protein (NVIE_004550 homolog)	X	X	X	X	X
NCAV_0488	Uncharacterized protein (NVIE_004540 homolog)	X	X	X	X	X
NCAV_0491	Uncharacterized protein (AmoX)	X	X	X	X	X

^a^Proteins with at least one AmoA, AmoB, or AmoC protein correlation.

^b^Conservation and exclusiveness to AOA based on results from Abby et al. (2020) [46].

^c^Correlation ≥0.7 and adjusted *p* value ≤ 0.001.

### Linkage analysis in AOA genomes supports proposed and additional AMO subunits

Earlier analyses of known subunits within the soil strains, or the family *Nitrososphaeraceae* (as defined by the Genome Taxonomy Database [47]; used throughout), has shown a general lack of spatial clustering of all earlier known subunit genes. However, within the families *Nitrosopumilaceae* and *Nitrosocaldaceae*, the genes for the canonical AMO subunits, AmoABC, and the proposed subunit AmoX are syntenic [36, 48, 49]. To investigate co-localization of potential additional subunit genes, the syntenic status and conservation across AOA of the five genes upstream and downstream of the *amo* gene cluster in *Nitrosocaldaceae* and *Nitrosopumilaceae* were analyzed. Of these genes, 19 were conserved in AOA with five being found exclusively in AOA (Supplementary Dataset 2). The five genes of interest included two canonical *amo* genes (*amoA* and *amoB*) and the genes *amoX*, NVIE_004540, and NVIE_004550. The absence of *amoC* in the genes of interest is attributed to a truncated version existing within the genome of “*Candidatus* Nitrosopumilus koreensis AR1” (likely due to assembly issues) that precluded it from being identified as conserved in all AOA. The *amoX* gene was previously identified in metagenomic studies [15, 36] and NVIE_004540 was already a candidate identified from the BN-PAGE correlation analysis. The additional conserved protein, NVIE_004550, was newly identified and found to be located directly upstream of NVIE_004540, indicating potential co-transcription. The two new candidates encode for polypeptides of 9.6 kDa and 12.8 kDa respectively, and – like the candidate subunit AmoX - their predicted secondary structure is predominantly helical and their subcellular localization transmembrane. The two new candidate *amo* genes NVIE_004540 and NVIE_004550 have therefore been termed *amoY* and *amoZ*, respectively.

**Fig. 3:**
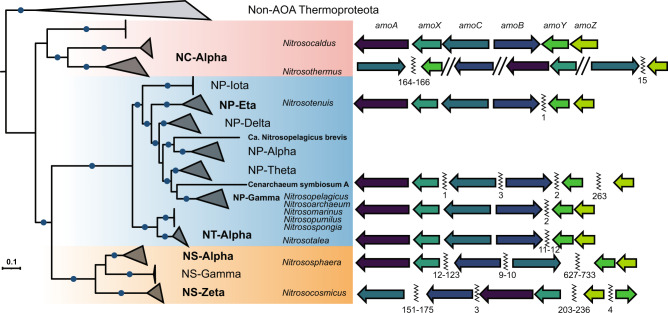
Genomic comparison of AMO subunit synteny in AOA. Left: Phylogenetic tree of AOA based on 32 conserved ribosomal proteins, ultrafast bootstrap values of 100% are indicated as blue circles. Taxonomic labels are colored according to GTDB family identity [47], *Nitrosocaldaceae*-red, *Nitrosopumilaceae*-blue, *Nitrososphaeraceae*-orange. Clades/organisms in bold were included in syntenic analysis. Clades are named according to Alves et al. (2018) [34]. Right: Representation of general syntenic patterns in different clades of AOA. Gaps between genes on the same contig are marked by a zig-zag line A double forward slash indicates separate contigs. Numbers under the zig-zag lines represent number of genes between *amo* subunit genes. A finer analysis and a full list of species can be found in Fig. S11 and Supplementary Dataset 2, respectively.

A closer analysis in *Nitrosocaldaceae*, the earliest diverging lineage in evolutionary reconstructions of AOA [46, 50], revealed that the genes for the three candidate subunits for AMO (AmoX, AmoY-homolog of NVIE_004540, and AmoZ-homolog of NVIE_004550) clustered spatially with the canonical subunits (AmoABC) and were syntenic in *Nitrosocaldus cavascurensis* and *Ca*. Nitrosocaldus islandicus. Spatial clustering of all six subunit genes is also found in recently obtained MAGs [51] within the genus *Nitrosocaldus*. In the case of the newly proposed genus *Ca*. Nitrosothermus [51], *amo* genes were split on multiple contigs and synteny could not be definitively determined (Fig. 3). Additionally, all six *amo* genes are inferred to have been newly acquired by the last common ancestor of AOA [46].

The emergence of *Nitrosopumilaceae* was accompanied by a separation of this genomic region into a primary cluster containing *amoABCX* and a secondary cluster containing *amoYZ* (Fig. 3). Within *Nitrosotalea* sp., these clusters are 11–12 genes apart, while the rest of *Nitrosopumilaceae* species have these clusters only 1–2 genes apart (with the exception of the sponge symbiont *Ca*. Cenarchaeum symbiosum). The emergence of the family *Nitrososphaeraceae* led to a scattering of all subunit genes across the genome with the exception of *amoA* and *amoX*, which are typically linked.

Although *amoZ* was identified in the genomic analysis, the protein AmoZ (NVIE_004550) was not correlated with AmoABC in the BN-PAGE gel of *N. viennensis*. When examining the relative abundance profile for AmoZ, the general pattern of AMO peptide peaks was followed. However, this remained undetected in the correlation analysis due to a high relative abundance peak occurring at the bottom of the gel peaking at the last band taken at approximately 66 kDa based on the BN-PAGE ladder (Fig. 1A). This is above the predicted mass of 12.8 kDa, but suggests that AmoZ could also be part of the AMO but a weaker association possibly lead to its dissociation from the complex and migration to the bottom of the gel.

### BN-PAGE protein gel indicates same AMO composition in the thermophilic archaeon Nitrosocaldus cavascurensis

To test the composition of the AMO complex outside of the context of *N. viennensis*, the BN-PAGE approach was applied to membrane protein fractions of *N. cavascurensis*, a distantly related thermophilic AOA species of the *Nitrosocaldaceae* family [48] that was recently obtained in pure culture (Melcher et al. in preparation). Although a slightly different pattern of complexes was obtained (Fig. 1B) a correlation of the additional subunits was also observed with AmoA, AmoB, and AmoC in this thermophilic organism (Kendall correlation of proteins, as performed for *N. viennensis*). The three proteins AmoX, NCAV_0488 (AmoY), and NCAV_0486 (AmoZ) all had migration patterns within the gel that strongly correlated with AmoABC (Table 1). This analysis confirmed that the proposed subunits were translated in *N. cavascurensis*, and potentially had a physical connection within the AMO complex.

### Chemical cross-linking supports physical interaction of additional subunits

**Fig. 4:**
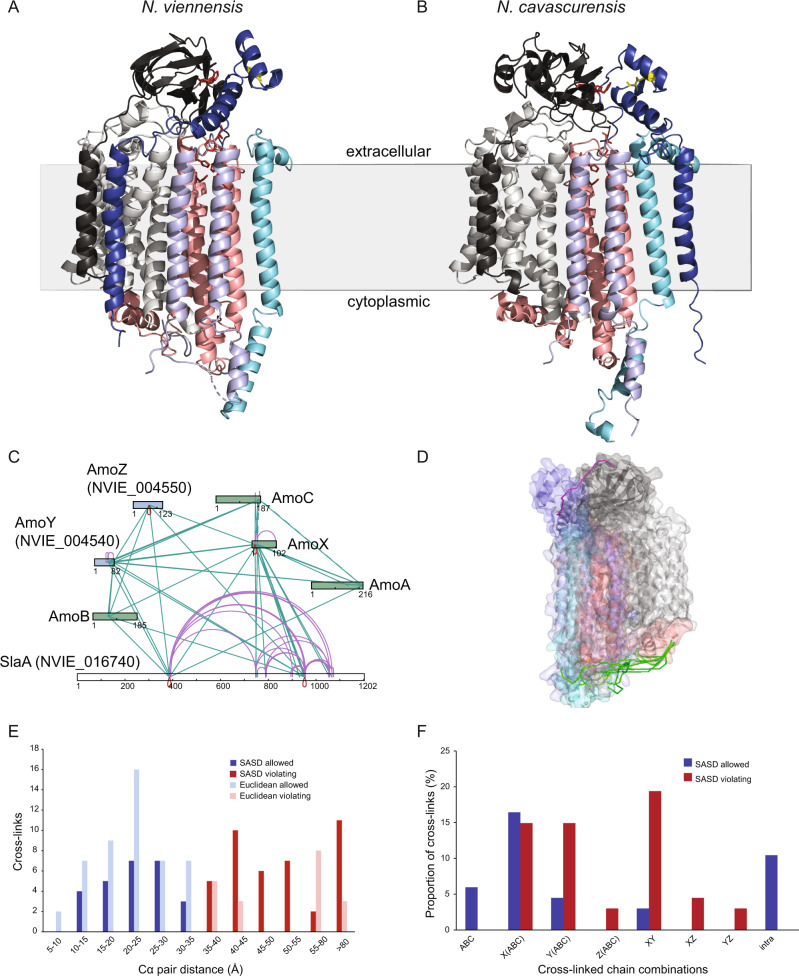
Structural support of proposed AMO subunits based on BN-PAGE cross-linking and AlphaFold modeling. **A**, **B** Cartoon representations of the AlphaFold structure models of the *N. viennensis* (**A**) and *N. cavascurensis* (**B**) hexamers, indicating their putative membrane orientation based on sequence hydropathy analysis. Subunits are colored as follows: AmoA, light grey; AmoB, black; AmoC, salmon; AmoX, lavender; AmoY, cyan; AmoZ, blue. Residues in the Cu_B_ and Cu_C_ copper sites are represented in red sticks. Disulfide bonds are indicated in yellow. **C** Representation of identified cross-links among existing and proposed AMO subunits of an AMO band cut from a BN-PAGE gel of *N. viennensis*. Green: suspected subunits based on comparative genomics. Blue: newly proposed subunits based on BN-PAGE correlation and syntenic analysis. **D** Cross-links within the solvent accessible surface distance (SASD) threshold for DSSO, depicted in green,  mapped on the *N. viennensis* AlphaFold model. The single observed cross-link between the AmoZ and AmoB subunits is depicted in magenta, as it violates SASD distance criteria (50.0 Å) but is within range of Euclidean distance (31.8 Å). **E** Distribution of SASD and Euclidean Cα–Cα distances of unique DSSO cross-links identified with Annika and MeroX. Twenty-seven out of 67 unique cross-links satisfied distance criteria (SASD < 35.0 Å). **F** Percentage of cross-linked subunit combinations.

To estimate the physical proximity of the proposed subunits to known subunits and other proteins within the BN-PAGE gel, in-gel cross-linking [43] was performed using the cross-linker disuccinimidyl sulfoxide (DSSO) on an additional BN-PAGE cut-out from band 7 (Fig. S1B). Mass spectrometry and cross-linking analysis showed multiple cross-links among AmoA, AmoB, AmoC, and AmoX as well as with the two newly proposed subunits AmoY and AmoZ (Fig. 4C). Many cross-links were also connected to NVIE_016740, a putative S-layer protein that likely represents a highly abundant surface layer protein as known from other archaea (SlaA) [52, 53]. As this protein presumably helps establish the pseudo-periplasm in AOA, it is not surprising to find it heavily cross-linked to membrane proteins.

AmoX also had individual cross-links to several other proteins (Supplementary Dataset 1). As only single connections were found, and these proteins did not appear in any other syntenic or correlative analyses, they were not taken to represent a structural role in the AMO complex. These cross-links can rather be attributed to the high abundance of those proteins in the cell membrane.

### Expression patterns of AMO subunits in Nitrososphaera viennensis and Nitrosopumilus maritimus

Available transcriptomic studies of AOA were inspected to explore whether the expression patterns of the newly predicted subunits would corroborate their involvement in the AMO. A recent study on copper limitation in *N. viennensis* [54] confirmed that the genes *amoA*, *amoB*, and *amoC* have some of the highest transcription levels in the cell, as also shown in previous studies [55–57]. A clustering analysis of the same dataset revealed that *amoA*, *amoB*, *amoC*, *amoX*, *amoY*, and *amoZ* all appear to be co-regulated, and fell into the clusters containing the most highly expressed genes. (Fig. S2; Supplementary Dataset 2). While putative transcriptional promoter sequences can be identified for most *amo* genes, an obvious conserved promoter motif for all six genes was not identified.

**Fig. 5:**
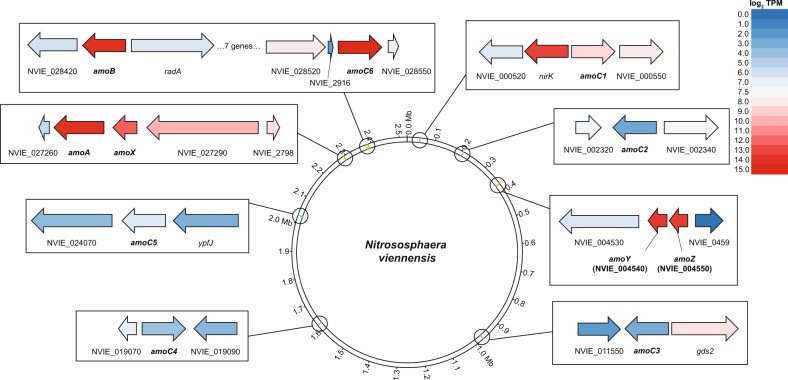
Transcription of AMO subunit genes in *N. viennensis*. Genomic representation of *N. viennensis* showing the location of *amo* genes and average log_2_ transformed transcript per million (TPM) values from copper replete conditions in Reyes et al. (2020) [54]. Orange bars on the genome indicate the locations of AMO subunit genes that are strongly expressed. Blue bars on the genome indicate *amo* genes that have low transcriptional activity. Boxes show *amo* genes (in bold) and immediate neighbors colored based on average gene expression from copper replete cultures. Red indicates a strong expression while blue represents a low or absent expression. All strongly expressed *amo* genes were found in clusters of highly expressed genes across both limited and replete conditions (see Fig. S2).

A re-evaluation of these transcriptomic data (see Methods) also revealed *amoC6* as the primarily transcribed *amoC* homolog (Fig. 5), thus confirming the identification of a unique AmoC6 peptide from a Tricine-SDS-PAGE band digested with chymotrypsin (Supplementary Discussion; Supplementary Dataset 1). Together this indicates that AmoC6 is the primary structural AmoC homolog in the AMO complex of *N. viennensis*, at least under the applied growth conditions.

Transcriptomics of the marine strain, *Nitrosopumilus maritimus*, also showed high expression of *amoA*, *amoB*, *amoC*, *amoX*, and *amoY* (Nmar_1506). The gene *amoZ* (Nmar_1507), albeit syntenic with *amoY*, exhibited lower expression levels [55].

The three newly proposed AMO subunits were also inspected in proteomic datasets that were generated with methods allowing for the improved recovery of membrane proteins. All six of the known and proposed subunits were found in membrane fractions from *N. viennensis* from a previous study [15] as well as in the proteome of *N. maritimus* [55]. In other proteomic studies of AOA [58, 59], the three new subunits were not always present, likely due to their small size and limited number of trypsin cleavage sites.

### Structural search for missing components in the archaeal AMO complex

As previously observed [60], comparisons of the amino acid sequences of the three subunits AmoA, AmoB, and AmoC from archaea with those of bacteria indicate that the primary differences between the archaeal AMO subunits and the bacterial AMO subunits are missing transmembrane helices, at least one in AmoB and two in AmoC, and a missing C-terminal soluble portion found in bacterial AmoB/PmoB (Figs. S3–S5). These observations also hold true for the new clade of archaeal AMO recently discovered in the Thermoplasmata phylum [61]. A HMMER search using the extended regions of the bacterial homologs against the genomes of collected AOA did not reveal any significant similarities. Therefore, a general structural search using Phobius [62] was carried out with the *N. viennensis* genome to search for genes that could encode a protein with the following criteria: (i) 1–3 transmembrane helices, (ii) conservation across all AOA [46], and (iii) present in the top 100 transcribed genes [54] (similar levels as the primary AMO subunits). This revealed six possible candidates (Table 2). The only candidates to meet the structural requirements while maintaining syntenic and similar patterns of migration in BN-PAGE were *amoX*, *amoY*, and *amoZ*.

**Table 2:** Structural search for missing AMO subunits in *N. viennensis*.

Gene Expression & Structural Search				Previous Analyses		Protein
Gene	TPM log2^a^	TM^b^	AOA Conservation^c^	BN-PAGE Corr.^d^	AMO Synteny^e^	
NVIE_013530	13.82	1		X		protein of unknown function
NVIE_004540	13.55	1	X	X	X	hypothetical protein
NVIE_004550	13.39	1	X	X	X	hypothetical protein
*amoX*	12.14	2	X	X	X	putative ammonia monooxygenase - associated protein
*coxB*	11.87	1		X		putative heme-copper oxidase subunit II
NVIE_014370	11.75	1				putative Copper binding protein, plastocyanin/azurin family
NVIE_010490	11.75	1				putative phosphoesterase, DHHA1
NVIE_027520	11.40	1	X			putative heme/copper-type cytochrome/quinol oxidase, subunit
NVIE_027550	11.33	1	X			putative blue (Type 1) copper domain protein
*atpK*	11.30	2				archaeal A1A0-type ATP synthase, subunit K
NVIE_021780	10.90	1				exported protein of unknown function
NVIE_006540	10.90	1				exported protein of unknown function
NVIE_029580	10.20	1	X			blue (Type 1) copper domain-containing protein

^a^Based on transcriptomic counts averaged from replete copper conditions from Reyes et al. (2020) [54].

^b^Number of predicted transmembrane helices.

^c^As predicted from Abby et al. (2020) [46].

^d^Represents proteins correlated with AmoA, AmoB, and AmoC6 in BN-PAGE bands from N. viennensis in this study.

^e^Genes syntenic with amoA, amoB, and amoC based on the syntenic analysis from this study.

The addition of the three proposed subunits in archaea increases the number of transmembrane helices from 10–11 to approximately 14 per protomer making it comparable to the number found in bacterial crystal structures of pMMO where each protomer of the trimer (i.e., one unit of PmoABC), contains 14–15 transmembrane helices [23, 63].

### Predicted structure of the archaeal AMO complex supports the integration of new subunits

To gain insights into the structural context of the archaeal AMO complex in the light of three additionally proposed subunits, a structural model for the organization of the *N. viennensis* AMO complex was obtained by employing the multimer-capable version of AlphaFold 2.1 [64–66]. The resultant models were all similar and represented confident predictions (top model, pLDDT = 71.4 and ptm score = 0.668). All predicted transmembrane helices from AmoX, AmoY, and AmoZ play a role in anchoring the complex in the membrane along with the transmembrane helices from AmoA, AmoB, and AmoC (Fig. 4A). Additionally, the N-terminal domain of AmoZ was predicted to contain two alpha helices that interact with the N-terminal domain of AmoB, thereby possibly replacing the role of the missing C-terminal soluble domain found in PmoB and offering the final piece of the missing complex in archaea (additional information in Supplementary Discussion). A disulfide bond was also predicted to form within the soluble domain of AmoZ. The overall structure is comparable to a protomer of the pMMO complex (Fig. S6).

To compare the degree of conservation of the predicted hexameric organization of the AMO complex, a structural model of the AMO complex of *N. cavascurensis* was also obtained (Fig. 4B). The resultant models were similar in their overall arrangement to each other and to the *N. viennensis* model, with high overall confidence scores (top model, pLDDT = 77.7 and ptm score = 0.591). Differences between the *N. viennensis* and *N. cavascurensis* models include the localization of the transmembrane (TM) helix of AmoZ. In *N. viennensis* the TM helix is predicted to interact mostly with the TM helix of AmoY, while in *N. cavascurensis* it is predicted to interact with the TMs of AmoB and AmoA (Figs. 4A, B, S7A, C). This would affect the relative positioning of the N-terminal domain of AmoZ with respect to the AmoB soluble domain, allowing for a more “open” conformation. However, the extended loop connecting the N-terminal pair of helices in AmoZ with the TM domain theoretically allows for some flexibility (additional information in Supplementary Discussion, Fig. S8).

Data from cross-linking experiments in *N. viennensis* were mapped to the predicted model and strongly supported the predicted interactions (Fig. 4D) with some exceptions. Out of 67 unique observed cross-links, 27 (40%) satisfied a maximum solvent accessible surface distance (SASD) threshold of ≤35 Å (Fig. 4E) and involved all subunit combinations with the exception of AmoZ (Fig. 4F). AmoZ only participated in cross-linking interactions >35 Å, which supports a weaker association with the complex as observed in the BN-PAGE migration patterns.

## Discussion

The archaeal AMO complex is a key enzyme of AOA energy metabolism that is highly expressed in all ammonia oxidizing organisms investigated and has large implications for the environment due to its overwhelming presence in many ecosystems [8–14, 67]. The work here profits from the recent improvements in the cultivation of AOA in continuous cultures (Melcher et al. in preparation) and presents novel biochemical and comparative genomic evidence on the composition of the AMO complex in *Nitrososphaera viennensis* and other AOA that contrasts with the proposed composition of this complex within AOB.

The present analysis has verified that AmoX, NVIE_004540, and NVIE_004550 are all likely present within the archaeal AMO complex and proposes the naming of NVIE_004540 and NVIE_004550 as AmoY and AmoZ, respectively. This finding is based on a host of independent analyses including proteomic, genomic, transcriptomic, structural, and modeling approaches. The presence of six subunits rather than three is unique to the archaeal domain and could represent a more complex regulatory strategy for the AMO complex in archaea. Differences in the ammonia oxidation pathway are already well established between the archaeal and bacterial domains (i.e., unresolved second step in archaea [19, 21]; iron-based *c* cytochromes [68, 69] and ubiquinone in bacteria [70, 71] vs. copper-based plastocyanins [15, 16] and menaquinone in archaea [72]). The varying characteristics within the AMO complex observed in this work further underscore these differences and add to a growing body of evidence that AOA and AOB participate in nitrification under different environmental and/or functional constraints.

In blue native PAGE protein gels, the AMO complex in both *N. viennensis* and *Nitrosocaldus cavascurensis* migrated well above the predicted height of a trimeric protomer complex, even when considering the additional subunits (predicted molecular weight of a homotrimeric complex with six subunits per protomer: 296.9 kDa *N. viennensis*; 305.1 kDa *N. cavascurensis*). The archaeal AMO bands are also observed at a higher molecular weight in the gel when compared to the homologous bacterial PMO complex from a *Methylomirabilis* species that was also extracted using n-dodecyl-β-D-maltoside (DDM) [73]. This could be explained by differences in membrane composition of the strains or potential differences in oligomerization of the protomer. AOA contain unique ether-linked lipids (i.e., crenarchaeols) [37, 74–78] and rely on a proteinaceous S-layer rather than an outer membrane to create a pseudo-periplasmic space [52, 53]. The observation of three distinct peaks of AMO can most likely be explained by the co-migration with other proteins or complexes that it could be physically interacting with, in particular with the S-layer protein.

Previous work on bacteria that rely on CuMMOs have identified other putative proteins involved with the complex. Monocistronic transcripts containing *amoABC* from the AOB *Nitrosococcus oceani* ATCC 19707 contained two additional genes assigned as *amoR* and *amoD* [79]. The *amoR* gene was found to be only present in *Nitrosococcus* and was therefore not thought to be a conserved part of bacterial AMO. A recent study indicated that AmoD/PmoD (and likely the homologous AmoE) play crucial roles in copper homeostasis, but they are not suspected to be a structural part of any CuMMO complex [80]. Rather, crystal and cryo-EM structures of bacterial PMO have consistently confirmed a trimeric protomer structure with one subunit of PmoA, PmoB, and PmoC making up each protomer [22–28].

Although there is debate on which subunit harbors the primary active site in CuMMO complexes, there is clear evidence that the metal site(s) in PmoC plays a critical role in the complex of methanotrophs [27, 28, 31, 32]. While the archaeal AmoC lacks a substantial section found in all bacteria that corresponds to two transmembrane helices (Fig. S5), the metal site observed in earlier crystal structures as well as the newly proposed metal site identified via cryo-EM [28]^,^ are conserved across all archaeal and bacterial species (Fig. S5). The importance of this subunit is also supported by site directed mutagenesis studies in the genetically tractable Actinobacteria that contain the homologous hydrocarbon monooxygenase [32].

The soil model AOA, *N. viennensis*, like most other soil dwelling AOA from the family *Nitrososphaeraceae*, encodes multiple homologs of the *amoC* gene while retaining only single copies of *amoA* and *amoB* [15] (Supplementary Dataset 2). Additional copies of *amoC* that are spatially disconnected from the AMO operon are encoded by some terrestrial AOB and were implicated in stress response based on transcriptional studies [81, 82]. Within *Nitrososphaeraceae*, no conserved AMO operons exist (Fig. 3). Duplications of the *amoC* gene (spatially distant from the other AMO genes) also occur in some species of the AOA marine associated family (*Nitrosopumilaceae)* and in two MAGs from AOA thermophiles (*Nitrosocaldaceae)*, all discovered in sediments [51, 83, 84]. An *amoC* duplication is also found in an AOA sponge symbiont and copies of archaeal *amoC* are even found in marine viruses [85]. These findings together may indicate the metabolic importance of the AmoC subunit for ecophysiological adaptations in ammonia oxidation. While this work identified one particular AmoC (AmoC6; NVIE_028540) to be the primary homolog within the complex of *N. viennensis*, it is possible that (some of) the other AmoC subunits, which arose by gene duplications at the genus level (Fig. S9), might be incorporated under certain environmental conditions and provide different activity profiles to the enzyme.

Beyond comparative genomics, the only confirmed structural information for archaea stems from the crystal structure of a heterologously expressed AmoB originating from *Candidatus* Nitrosocaldus yellowstonensis [86]. This structure confirmed the lack of the C-terminal cupredoxin domain and revealed an extended amino acid region not found in bacteria made up of two helices and two loops. It was proposed that this additional region could help stabilize the existing cupredoxin domain as supportive interactions are lacking due to the absence of the C-terminal domain. However, this amino acid extension is only found within the proposed genus of *Nitrosocaldus* (Fig. S4). It is more likely that the soluble domain of AmoZ, which is conserved across all AOA, is conferring this stabilizing role.

In the absence of additional structure-function analyses, it is unclear if the additional subunits in the archaeal complex simply reflect the vast evolutionary distance to all other known protein complexes of the CuMMO family [34], or if this difference in structure also has relevant functional implications. For instance, the bacterial AMO complexes are promiscuous enzymes able to oxidize methane and other compounds [87–90]. Such investigations on alternative substrates have not yet been performed with the archaeal complex but would be important for evaluating the functional role of archaea (and possibly the new subunits) in the environment. Additionally, differences in the AMO complex between AOA species have been identified that have the potential to affect the function of the AMO complex. This includes the extra AmoB loop found within *Nitrosocaldus*, but is also clearly demonstrated by the newly proposed subunit AmoZ. The genus *Nitrososphaera* and the family *Nitrosocaldaceae* (both investigated in this study) are predicted to form a disulfide bond linking the two alpha helices making up the soluble domain of AmoZ (Figs. S7B, D, S10C) via two cysteines that are not conserved in other AOA. Additionally, the genus *Nitrosocosmicus* is predicted to contain an additional zinc ribbon domain represented by four cysteine residues at the C-terminal end, presumably residing within the cytoplasm (Fig. S10C). The observations of a disulfide bond and zinc ribbon domain within certain AOA lineages could be linked to sensitivity to reactive oxygen species and unique regulation strategies, respectively, and may reflect unique patterns of evolution that complement yet unknown aspects of the metabolism of these specific groups.

Regardless of species-specific differences in archaea, the overall predicted archaeal structure, with the new subunits, is reflective of the known bacterial protomer composition (Fig. S6). Definitive proof of the oligomerization and organization of these subunits will not be possible until a definitive structure (i.e., crystal or cryo-EM) of archaeal AMO is realized. Putative protomer interaction sites in the cryo-EM structure of *Methylococcus capsulatus* str Bath (PDB structure 7S4H) [28] appear to be in the section of PmoB that is missing in archaea (Fig. S4). However, the placement of AmoY and AmoZ on the outer regions of the protomer could be facilitating these interactions instead (Fig. S7). This could also explain the high amount of SASD violating interactions between AmoY and AmoZ as the analysis only takes into account a single protomer (Fig. 4F). It is possible that these interactions may instead be between subunits of AmoY and AmoZ in different protomers. Therefore, the predicted protomer models of *N. viennensis* and *N. cavascurensis* do not rule out the possibility of a trimeric archaeal AMO complex. With respect to orientation, the present modeling approach is not able to predict exactly how the archaeal AMO sits in the membrane. However, it is likely that the active site as well as the soluble AmoB and AmoZ domains are situated toward the pseudo-periplasmic space. This is supported by previous modeling efforts of nutrient transport in the S-layer of AOA [91] as well as activity-based immunogold labeling of CuMMO complexes in AOB and methanotrophs [92].

In conclusion, this study provides evidence through genomic, proteomic, and transcriptomic data for the presence of AmoX and the inclusion of AmoY and AmoZ as subunits within the archaeal AMO complex. A single protomer of the archaeal AMO would therefore consist of six subunits instead of three as in other complexes of the CuMMO family. The addition of the new subunits would make the number of transmembrane helices comparable to CuMMO complexes found within bacteria. As the anchoring of pMMO in the membrane has previously been shown to be critical for its activity [26, 28], it seems plausible that the newly identified subunits play an important role for the structural and functional integrity of the archaeal AMO complex. The presence of a soluble domain within AmoZ that could replace the stabilizing function of the missing soluble domain in AmoB also fulfills a potentially crucial missing piece of the AMO complex. Since AmoXYZ appear to have important structural roles, it will be imperative to include all subunits in future expression and structural studies of this environmentally relevant protein complex in archaea. Considering the wide distribution of AOA in virtually all ecosystems [8–14, 34] and their ecological relevance, developing genetic tools for AOA and improving their biomass production will be needed to enable structure-function analysis and to elucidate the full pathway of ammonia oxidation in these archaea.

## Supplementary information

Supplementary Material

Dataset S1

Dataset S2

Dataset S3

Dataset S4

Dataset S5

Dataset S6

Dataset S7

Dataset S8

## Data Availability

All proteomic data was deposited to the ProteomeXchange Consortium via PRIDE [44] partner repository with identifiers PXD035349, PXD034632, and PXD034475 for BN-Page of N. viennensis, BN-PAGE of N. cavascurensis, and cross-linked samples, respectively. Relevant scripts and code for data analysis can be found at the GitHub repository https://github.com/hodgskiss/Archaeal_AMO.
