# Correction to: Unexpected complexity of the ammonia monooxygenase in archaea

**DOI:** 10.1038/s41396-023-01403-2

**Published:** 2023-04-28

**Authors:** Logan H. Hodgskiss, Michael Melcher, Melina Kerou, Weiqiang Chen, Rafael I. Ponce-Toledo, Savvas N. Savvides, Stefanie Wienkoop, Markus Hartl, Christa Schleper

**Affiliations:** 1 Archaea Biology and Ecogenomics Unit, Department of Functional and Evolutionary Ecology, University of Vienna, Vienna, Austria; 2 Mass Spectrometry Facility, Max Perutz Labs, Vienna BioCenter (VBC), Vienna, Austria; 3 VIB Center for Inflammation Research and Department of Biochemistry & Microbiology, Ghent University, Ghent, Belgium; 4 Molecular Systems Biology Unit, Department of Functional and Evolutionary Ecology, University of Vienna, Vienna, Austria; 5 Department of Biochemistry and Cell Biology, Max Perutz Labs, University of Vienna, Vienna, Austria

**Keywords:** Archaeal physiology, Soil microbiology, Metabolism, Environmental sciences, Proteomics

Correction to: *The ISME Journal* 10.1038/s41396-023-01367-3, published online 31 January 2023

In this article the affiliation details for Savvas N. Savvides were incorrectly given. It should have been ‘VIB Center for Inflammation Research and Department of Biochemistry & Microbiology, Ghent University, Ghent, Belgium’.

The original article has been corrected.

