# Supplementary material for: Unexpected complexity of the ammonia monooxygenase in archaea
Source: ISME J. 2023 Jan 31;17(4):588–99. doi: 10.1038/s41396-023-01367-3 (PMC10030591; doi:10.1038/s41396-023-01367-3)
Supplement: Supplementary file 1 — Supplementary Material [file 41396_2023_1367_MOESM1_ESM.pdf]

Supplementary Information for

Unexpected Complexity of the Ammonia Monooxygenase in Archaea

Logan H. Hodgskiss[1], Michael Melcher[1], Melina Kerou[1], Weiqiang Chen[2], Rafael I. Ponce-Toledo[1], Savvas N. Savvides[3], Stefanie Wienkoop[4], Markus Hartl[2,5], & Christa Schleper[1*]

[1]Archaea Biology and Ecogenomics Unit, Department of Functional and Evolutionary Ecology, University of Vienna
[2]Mass Spectrometry Facility, Max Perutz Labs, Vienna BioCenter (VBC), Austria
[3]Unit for Structural Biology, Department of Biochemistry and Microbiology, Ghent University, Ghent, Belgium
[4]Molecular Systems Biology Unit, Department of Functional and Evolutionary Ecology, University of Vienna
[5]Department of Biochemistry and Cell Biology, Max Perutz Labs, University of Vienna, Austria

*Corresponding author: Christa Schleper

Email: christa.schleper@univie.ac.at

This PDF file includes:

Legends for Datasets S1 to S4
Supplementary Discussion
Supplementary Materials and Methods
Figures S1 to S12
Supplementary Information References

Other supplementary materials for this manuscript include the following:

Datasets S1 to S8

Legends for Supplementary Datasets

Dataset S1. (separate file)
Excel sheet containing proteomic data and supplementary tables.

Dataset S2. (separate file)
Excel sheet containing genomic and transcriptomic data and supplementary tables.

Dataset S3. (separate file)
A .pdb file for the predicted AlphaFold2.1 structure of AMO in *Nitrososphaera viennensis* (Alphafold_NvAMO_rank_1.pdb).

Dataset S4. (separate file)
A .pdb file for the predicted AlphaFold2.1 structure of AMO in *Nitrosocaldus cavascurensis* (Alphafold_NcavAMO_rank_1.pdb).

Dataset S5. (separate file)
PyMOL session file for AMO model of *Nitrososphaera viennensis* as seen in Figure 4A (Nvie_Fig4A).

Dataset S6. (separate file)
PyMOL session file for AMO model of *Nitrosocaldus cavascurensis* as seen in Figure 4B (Ncav_Fig4B).

Dataset S7. (separate file)
PyMOL session file for AMO model of *Nitrososphaera viennensis* overlayed on the PMO crystal structure (PDB:7S4H, pMMO) from *Methylococcus capsulatus* (Bath) as seen in Figure S6C (Nvie_PMO_overlay_FigS6C).

Dataset S8. (separate file)
PyMOL session file for AMO model of *Nitrososphaera viennensis* overlayed on the AMO model of *Nitrosocaldus cavascurensis* as seen in Figure S6D (Nvie_Ncav_overlay_FigS6D).

Supplementary Discussion

Tricine-SDS-PAGE verifies components of the ammonia monooxygenase complex

To further explore the content of the ammonia monooxygenase (AMO) complex extracted from BN-PAGE, cut-outs from band 7 (Fig. 1A) were subjected to Tricine-SDS-PAGE analysis under denaturing conditions [1, 2]. Tricine-SDS-PAGE gels were stained with two silver staining methods and a method using SimplyBlue SafeStain. As silver staining methods can interfere with mass spectrometry analysis, silver staining methods were used for better visualization of bands, while the SimplyBlue SafeStain was used to allow for better extraction of proteins for mass spectrometry proteomic analysis. Tricine-SDS-PAGE bands containing AmoA, AmoB, AmoC, and AmoX were all identified using trypsin as the digestive enzyme (Fig. 2). Apparent sizes of AmoB, AmoC, and AmoX proteins matched the expected molecular weight, while the band representing AmoA had a lower apparent molecular weight than expected, likely due to the high hydrophobicity of this subunit. Proteins with multiple transmembrane helices have been observed to run faster than expected in SDS-PAGE gels [3]. Bands containing SlaA (S-layer protein), NVIE_028570 (exported protein of unknown function), and NVIE_024150 (ABC uptake transporter) were also identified. The band containing AmoB also contained a large amount of NVIE_021780 (exported protein of unknown function) and the one containing the highest amount for AmoX was dominated by the two hypothetical proteins identified from the syntenic analysis, NVIE_004540 and NVIE_004550, which are proposed here to be part of the archaeal AMO complex as AmoY and AmoZ, respectively. The band representing AmoC could be separated into two primary protein groups: one representing amoC4/C6 that made up 96% of the total AmoC intensities, and one representing amoC1/C2 at 4%. While unique peptides were identified for AmoC3, they represented less than 0.05% of total AmoC intensities.

In an attempt to obtain unique AmoC peptides to distinguish AmoC4 and C6, which are 96 % identical in their amino acid sequence (BLASTp) [4, 5], additional replicate cut-outs from BN-PAGE gels corresponding to band 7 were subjected to SDS-Tricine-PAGE. Bands corresponding to AmoC were digested using two different proteases: GluC endoprotease or chymotrypsin. After chymotrypsin digestion it was

possible to identify unique peptides from AmoC6, although the majority of AmoC peptides came from a shared region of both peptides. Peptide coverage maps for GluC, chymotrypsin, and trypsin digestion for the six homologs can be found in Supplementary Dataset 2. A unique peptide was not found for AmoC4 after correction of its predicted translational start site based on available transcriptomic and proteomic data (see Materials and Methods below). Therefore, it was not possible to definitively distinguish in these experiments between C4 and C6 as the dominant homolog in the AMO complex with proteomics alone. However, this data combined with transcriptomic data from Reyes et al. (2020) [6] identifies AmoC6 as the primary homolog in *Nitrososphaera viennensis*.

Architecture of the structural model of AmoABCXYZ in Nitrososphaera viennensis *and* Nitrosocaldus cavascurensis *and notes on the putative role of the novel subunits*

The AlphaFold multimer model of the *N. viennensis* AMO complex comprises single copies of the AmoA, AmoB, AmoC, AmoX, AmoY, and AmoZ subunits (Fig. 4, Fig. S7). Subunits AmoB and AmoZ contained predicted signal peptides which were not included in the modeled sequences. AmoA consists of six transmembrane (TM) helices and is encircled by the TM helices from AmoC, AmoB, AmoX and AmoZ, forming the core of the TM region of the complex. A loop and short helix formed between helices five and six protrude from the membrane and interact with the soluble domain from AmoB, as in the pMMO structure. AmoB consists of an N-terminal soluble β-barrel formed by seven antiparallel strands and a C-terminal TM helix which interacts with TM helices from AmoA and AmoZ. AmoC consists of four TM helices encircled by the helices of AmoA, AmoY, and AmoX, and a pair of short C-terminal helices oriented perpendicular to the helices of AmoA on the cytoplasmic side of the molecule. AmoX consists of two TM helices and an N-terminal loop and short helix extending towards the cytoplasmic side of the complex, interacting putatively with the cytoplasmic section of AmoC. AmoY forms a single TM helix interacting with AmoC, with shorter helices extending towards both extracellular and cytoplasmic sides. In addition to the stabilizing role of their TM helices for the holoenzyme, both AmoY and AmoZ encode cytoplasmic helices which could be important for protein interactions as part of signaling cascades on the cytoplasmic side.

The proposed subunit AmoZ contains a soluble N-terminal domain formed by an extended disordered region and two alpha helices connected to a C-terminal transmembrane domain by an extended loop. The two alpha helices are stabilized by a disulfide bond between Cys 49 and Cys 59, conserved in the genus *Nitrososphaera* and in the family *Nitrosocaldaceae* (Fig. 4, Fig. S7A,C, Fig. S8B, D, Fig. S10C). A hydrogen bond between Glu 48 and Arg 68 offers additional stabilization of the two helices (Fig. S8B). Glu 48 is conserved in all AmoZ homologs, while the position of Arg 68 can also be occupied by a lysine or a glutamic acid. Interactions with the cupredoxin domain of AmoB can be observed in the form of hydrogen bonds between AmoZ-Arg 69 and AmoB-Glu 83 (Fig. S8B). Both sites are universally conserved, corroborating the putative stabilizing role of the AmoZ. The amino acids Glu 61 in *N. viennensis* and Gln 60 in *N. cavascurensis* are in close vicinity to the conserved AmoB histidines (His 126 and His 128 in *N. viennensis*) coordinating the Cu_B metal site (Fig. S8D), but it is unclear whether this affects the ionic environment of the metal site.

The Cu_C copper site in the *N. viennensis* model exhibits a few putative differences compared to the pMMO site. In addition to the canonical coordination residues Asp 41, His 45, and His 58 from AmoC, the conserved N-terminal histidine from AmoB (His 31) is modelled as part of the coordination sphere, within 3Å of the previous residues (Fig. S8A). In contrast, the amino-terminal histidine is part of the Cu_B center in pMMO. In the *N. cavascurensis* model, the flexibility of the N-terminal region of AmoB could result in the same orientation. The N-terminal tyrosine of AmoZ (Tyr 31) in *N. viennensis* is modelled in close vicinity (within 4.5-7.2 Å) to the Cu_C coordination sphere (Fig S8A). Since this residue is universally conserved, implying an essential role, and the N-terminus of AmoZ is a flexible region, it is interesting to speculate whether it can influence the charge distribution in the vicinity of this metal site in archaea. The identification of a potential new metal site within PmoC from *Methylococcus capsulatus* (Bath), termed Cu_D, was recently identified using cryo-EM structures in native membrane nanodiscs [7]. This new metal site, likely containing copper, is coordinated by two histidines and an asparagine within the PmoC subunit. These amino acids are conserved across all species containing copper membrane bound monooxygenases, including archaea (Fig. S5). The addition of this newly conserved metal site strengthens the importance of the

PmoC/AmoC subunit within the PMO/AMO complex as it is the only subunit with universally conserved metal binding sites.

The predicted models of *N. viennensis* and *N. cavascurensis* are similar in their general architecture, with a few notable differences (Fig. S6D). The relative positioning of AmoZ is discussed in the main text, and due to its overall weaker interactions with the complex it is evident that high-confidence modeling of this subunit is not possible with current methods, as shown also by its overall lower confidence score in the models (Fig. S7B,D). The AmoB subunit of *N. cavascurensis* encodes an extended section of the N-terminal extracellular domain between β strands 1 and 2 (residues 55-81), which forms two short helices and two loops and is anchored to the core β-barrel domain with a disulfide bond between Cys 76 and Cys 140 and multiple hydrogen bonds (Fig. S7C, S8C), as already observed by Lawton et al. (2014) [8] in the crystal structure of AmoB from "*Candidatus* Nitrosocaldus yellowstonensis". The putative role of this extension, which is only found in the genus *Nitrosocaldus*, could be an enhanced stability of the enzyme in the high temperatures experienced by this lineage. However, this would not explain the lack of this extension within the newly proposed thermophilic genus "*Ca.* Nitrosothermus".

Superimposition of the *N. viennensis* model to the cryo-EM structure of the particulate methane monooxygenase (PDB:7S4H, pMMO) from *M. capsulatus* (Bath) (cealign in PyMOL), reveals conservation of the overall fold of the main subunits AmoA, AmoB and AmoC, with an RMSD of 3.382 Å over 200 residues (Fig. S6A,B,C). Known differences include the absence of the second cupredoxin domain and the second TM helix of PmoB in AmoB, absence of the seventh TM helix of PmoA in AmoA, and the different localization of AmoC in the multimer compared to PmoC, which in part accommodates the two TM helices of AmoX.

<u>Supplementary Materials and Methods</u>

AMO Alignments for AmoABC

Fifty archaeal species' and 29 bacterial species' genomes were collected and searched for known AMO/PMO subunits (AmoA/PmoA, AmoB,/PmoB and

AmoC/PmoC). Full lists of the collected species can be found in Supplementary Dataset 2.

If a species was not annotated, genomic.fna files were collected from NCBI and coding sequences were searched for using prodigal (version 2.6.3) [9] using the parameter "-p single". In the case of annotated species, RefSeq annotation files were given preference if available. When no RefSeq annotation was available, GenBank annotations were used. In the case of "*Candidatus* Cenarchaeum symbiosum A", the genome file was re-annotated using prodigal. This was done to search for coding regions that should theoretically be present that were not detected in the given annotation file.

Hidden Markov Models (HMMs) were made for archaea and bacteria separately based on amino acid sequences of well documented species with representatives from all major clades. In archaea, sequences from *Nitrososphaera viennensis* EN76 (*Nitrososphaeraceae*), *Nitrosocaldus cavascurensis* (*Nitrosocaldaceae*), *Nitrosopumilus maritimus* SCM1 (*Nitrosopumilaceae*, formerly Nitrosopumilales), and "*Ca.* Nitrosotalea devaneterra" (*Nitrosopumilaceae*, formerly Nitrosotaleales), were chosen to construct the model. In bacteria, sequences from *Nitrosococcus oceani* ATCC 19707 (Gammaproteobacteria, ammonia oxidation), *Nitrosospira multiformis* ATCC 25196 (Betaproteobacteria, ammonia oxidation), "*Ca.* Nitrospira inopinata" (Nitrospira, comammox), *Methylosinus trichosporium* OB3b (Alphaproteobacteria, methanotrophy), *Methylococcus capsulatus* str. Bath (Gammaproteobacteria, methanotrophy), *Methylacidiphilum kamchatkense* Kam1 (Verrucomicrobia, methanotrophy), and *Mycolicibacterium chubuense* NBB4 (Actinobacteria, hydrocarbon oxidation), were chosen to construct the model.

Sequences from representative species of archaea and bacteria were aligned using Mafft (version 7.427) [10, 11] and an HMM model was constructed using hmmbuild (HMMER 3.3, hmmer.org) for each subunit in archaea and bacteria separately. An HMM search using hmmsearch (HMMER 3.3) was performed on selected species and sequences were collected for archaea and bacteria. Archaeal and bacterial species were searched separately due to the distant phylogenetic relationship of the AMO/PMO complex within the two domains. A cut off value of 1e-20 was used for annotated species

while a cut off value of 1e-10 was used for species analyzed with prodigal. A lower threshold was used for un-annotated species to account for the possibility of partial AMO/PMO genes on the edge of contigs. While a genome is not available for *Methylocystis* sp M., sequences were added to the appropriate bacteria files after the hmmsearch. Once collected, archaeal and bacterial sequences were combined and aligned using Mafft with the "mafft-linsi" parameter. Sequences that clearly did not belong after the multiple sequence alignment (due to the presence of stop codons or inclusion due to the low threshold) were manually removed (two in the case of bacterial AmoB/PmoB).

Phylogenetic tree of archaeal amoC *genes*

The archaeal *amoC* tree was constructed from a nucleotide BLAST (blastn, BLAST 2.12.0+) [12] search using sequences from AOA species representing the dominant clades of AOA (see above). Mafft was used to align the nucleotide sequences. The alignment was then trimmed using BMGE (v1.12) [13] using parameter "-g 0.3" and IQTree (version 2.1.2) [14] was used to construct the phylogeny of archaeal *amoC* using 1000 ultrafast bootstrap replicates [15]. Nucleotide sequences were used for the tree construction as amino acid sequences were too similar to construct a reliable phylogeny.

Phylogenomic analysis

A total of 106 MAGs and completely sequenced genomes (98 AOA and eight non-AOA genomes) were collected from NCBI, IMG, or DDBJ databases, followed by protein prediction using Prodigal v2.6.3 [9]. The identification of phylogenetic markers to perform the phylogenomic tree reconstruction was based on the workflow proposed by Graham et al. (2018) [16] using the archaeal single-copy gene collection (e-value 10^{-10}) [17]. Thirty-two ribosomal proteins detected in at least 90 out of the 106 genomes present in the collected genome database were selected. Protein families were aligned independently using the mafft-linsi algorithm implemented in MAFFT v7.427 [11] followed by a trimming step in BMGE [13] with default parameters. Trimmed protein families were concatenated using a tailormade python script and the concatenated

alignment was used to reconstruct a maximum likelihood (ML) phylogenomic tree in IQTREE (v2.0-rc1) [14] under the LG+C20+F+G model with 1000 ultrafast bootstrap replicates [15].

Membrane protein extraction

Procedures for protein extraction and running a BN-PAGE gel were based off of previous studies [18, 19] and the NativePAGE Novex Bis-Tris Gel System manual from Life Technologies (MAN0000557). Study design and analysis for membrane extraction and BN-PAGE was also largely guided by previous studies [20, 21].

Frozen pellets of biomass were thawed on ice and resuspended in a sodium phosphate buffer solution (50 mM sodium phosphate, 200 mM NaCl, pH 7.0) to a concentration of ~20 mg/mL. Once resuspended, pepstatin and Complete Tablet EDTA-free inhibitor solution were added at concentrations of 1 µg/mL and 40 µL/mL (25x concentrated stock), respectively, to inhibit protease activity. Cells were lysed using a One Shot machine set at 2.1 kbar of pressure. After lysis, samples were spun at 7 000 x g for 15 minutes at 4 °C to remove cellular debris. The supernatant was then taken for further processing.

Supernatant containing proteins and membrane were ultracentrifuged at 200 000 x g (Beckman Coulter Ultracentrifuge; SW 41 Ti Swinging-Bucket Rotor, k_{max}=124) for 90 minutes at 4 °C using 13.2 mL thinwall polypropylene tubes with a level of deceleration set to 7. The supernatant (containing cytoplasmic proteins) was removed and stored at -70 °C with 10% glycerol. The remaining membrane pellet was resuspended/washed in a solution of 1 M NaCl, 50 mM Tris-HCl, pH 7.5 and then concentrated via ultracentrifugation at 200 000 x g for 90 minutes at 4 °C. The supernatant was again removed and stored at -70 °C with 10% glycerol. A final washing of the membrane fraction was performed by resuspending the pellet in 50 mM Tris-HCl, pH 7.5 and then concentrated via ultracentrifugation at 200 000 x g for 90 minutes at 4 °C. (Important: Washing solutions are made with Tris-base and titrated with HCl to avoid accumulation of sodium ions that interfere with BN-PAGE gels. Do not make with Tris-HCl and titrate

with NaOH.) After the final wash the supernatant was again removed and stored at -70 °C with 10% glycerol.

The final membrane pellet was resuspended in 100-200 µL of NativePage Sample Buffer (Invitrogen BN2003) with 0.75 M of 6-aminocaproic acid. To aid in the resuspension of membrane fractions, samples were allowed to gently mix on a rotator set at 12 rpm at 4 °C for 30 minutes. Following the incubation on the rotator, protein concentrations were measured using the Bradford assay (Bio-Rad #5000006). Based on the protein concentration, n-dodecyl-β-D-maltoside (DDM, Invitrogen BN2005) was added to the sample at a concentration of 0.5 g DDM/g protein. Samples were again incubated on a rotator at 12 rpm and 4 °C for 30 minutes. A final protein concentration was determined using the Bradford assay (Bio-Rad #5000006) with a bovine serum albumin (BSA) standard curve and controls to account for interference from DDM. Samples were then aliquoted into volumes containing approximately 40-50 µg of protein and frozen at -70 °C for later analysis.

Blue Native PAGE

Frozen membrane samples (40-50 µg aliquots; ~10-20 µL) stored at -70 °C were thawed on ice. Based on previously calculated protein concentrations, additional DDM was added to each sample to reach a DDM concentration of 0.75 g DDM / 1 g protein. Samples were then incubated in a shaker at 700 rpm for 15 minutes at 4 °C. After incubation, samples were centrifuged at 9 000 x g for 60 minutes at 4 °C to remove any cellular debris that was not solubilized by the addition of DDM. Supernatant from this centrifugation was transferred to a 1.5 mL LoBind protein tube (Eppendorf). Coomassie (NativePage 5% G-250 Sample Additive, Invitrogen BN2004) was added to each sample to reach a Coomassie:DDM ratio of 1:1 (w/w). Samples were then loaded on a 3-12% pre-cast BN-PAGE gel (Invitrogen BN1001). Approximately 5-7 µL of NativeMark Unstained Protein standard (Invitrogen LC0725) was used as the ladder. The anode buffer was pre-ordered (Invitrogen BN2001) and consisted of a final concentration of 50 mM Bis-Tris and 50 mM tricine at pH 6.8. The gel was run at 4 °C in three stages. The first stage used dark blue cathode buffer (anode buffer with cathode buffer additive (Invitrogen BN2002): 50 mM Bis-Tris, 50 mM tricine, 0.02% Coomassie G-250, pH 6.8)

and was run for 1 hour at 150 V. For the second stage, the dark blue cathode buffer was replaced with light blue cathode buffer (50 mM Bis-Tris, 50 mM tricine, 0.002% Coomassie G-250, pH 6.8) and the gel was run for an additional hour at 250 V. For the third and final stage, the light blue cathode buffer was replaced with anode buffer and run for 45 minutes at 250 V.

When finished, gels were stained using SimplyBlue SafeStain (Invitrogen LC6060; maximum sensitivity protocol). Destaining was done with MilliQ (MilliporeSigma Milli-Q Reference A+ System) water and repeated until as much background could be removed as possible. Individual bands identified in each gel were removed and cut into 2-3 pieces and placed into LoBind protein Eppendorf tubes. Bands were stored at 4 °C in 150 µL of MilliQ water until being processed for proteomic analysis, cross-linking, or SDS-Tricine-PAGE.

Tricine-SDS-PAGE

Procedures for running an Tricine-SDS-PAGE gel were primarily based on previous studies [1, 2]. A 15% Tricine-SDS-PAGE gel was made by mixing: 2.5 mL 30% acrylamide/Bis solution 37.5:1 (BioRad #1610158), 1.25 mL gel buffer (1.5 M Tris-HCl, 8.45 pH), 1.15 mL water, 50 µL 10% sodium-dodecyl sulfate (SDS), 50 µL 10% ammonium persulfate (APS), and 5 µL 1,2-bis(dimethylamino)ethan (TEMED). Once the gel solidified a 4% stacking gel consisting of 340 µL 30% acrylamide/Bis solution 37.5:1, 250 µL gel buffer (1.5 M Tris-HCl, 8.45 pH), 1.36 mL water, 20 µL 10% SDS , 20 µL 10% APS , and 2 µL TEMED was poured on top. Selected BN-PAGE bands were cut into 2-3 pieces and incubated together in 20 µL SDS loading buffer (0.2 M Tris-HCl, 0.3 M dithiothreitol (DTT), 277 mM SDS (8% w/v), 6 mM bromophenol blue, 4.3 M glycerol) at 65 °C for 90 minutes in a shaker at 500 rpm. The loading dye solution from this incubation was used to load the Tricine-SDS-PAGE gel. Either 3-5 µL of Color Prestained Protein Standard-Broad Range (11-245 kDa) (New England Biolabs, P7712) or 1-3 µL of PageRuler Prestained Protein Ladder (10-180 kDa) (Thermo Scientific, 26617) were used as a ladder. Stock solutions of 10x concentrated anode buffer (1 M Tris-base, adjusted with 6 M HCl to a pH of 8.9) and 10x concentrated cathode buffer (1 M Tris-base, 1 M tricine, 1% SDS, pH 8.3 (no pH adjustment necessary)) were

previously made. Buffers were diluted to 1x when used for running the gel. The gel was run at 30 V for 25 minutes to allow the proteins to leave the stacking gel followed by 200 V for ~55 minutes or until the ladder reached the bottom of the gel. For clear visualization, Tricine-SDS-PAGE gels were silver stained (see below). If the bands were to be used for proteomic analysis, gels were stained with SimplyBlue SafeStain (maximum sensitivity protocol). After staining, bands were cut from the gel and placed in 1.5 LoBind protein tubes (Eppendorf) with 150 µL of MilliQ water and stored at 4 °C until being processed for proteomic analysis.

Silver staining of Tricine-SDS-PAGE gels

All steps were done using a gentle shaker. Gels were soaked in fixing solution (50% methanol, 12% acetic acid) for at least one hour to overnight. Containers with gels fixed overnight were sealed with parafilm to prevent evaporation. Gels were washed for 20 minutes in 50% ethanol. Washing was repeated twice for a total of three times. The gel was then soaked for one minute in a freshly prepared solution of 1.2 g/L of sodium thiosulfate pentahydrate. Next the gel was washed for 30 seconds in MilliQ water. This was repeated twice for a total of 3 times. After washing, the gel was soaked in the dark in freshly prepared silver staining solution (2 g/L silver nitrate, 0.04% formaldehyde) for 25-30 minutes. Following staining, the gel was washed twice with MilliQ water for 30 seconds. To develop bands, the gel was submerged in developer solution (60 g/L sodium carbonate, 0.04% formaldehyde, 0.036 g/L sodium thiosulfate pentahydrate). Bands developed within 1-3 minutes and development was stopped by adding destain solution (10% acetic acid, 1 % glycerol). The gel was then soaked in destain solution for approximately five minutes before being washed multiple times with MilliQ water.

Silver staining (Farmer's Reducer) of Tricine-SDS-PAGE gels

The gel was soaked in fixing solution as stated above. Fixing solution was removed and the gel was soaked in Farmer's Reducer (30 mM $K_3Fe(CN)_6$, 30 mM sodium thiosulfate pentahydrate) for 2 minutes. This turned the gel yellow. The gel was then washed multiple times with MilliQ water until the yellow background was completely removed (30-90 minutes). Once the background was removed, water was

poured off and replaced with 0.1% silver nitrate and was incubated in the dark for 15 minutes. After incubation, the gel was washed multiple times with MilliQ water. The gel was then submerged in a 2.5% sodium carbonate solution for 30 seconds. To develop bands, the solution was removed and replaced with a solution of 0.1% formaldehyde and 2.5% sodium carbonate. Development was stopped with destain solution as described above.

DSSO cross-linking of Blue Native PAGE cut-outs

The protocol from Hevler et al. was followed [22]. To summarize, BN-PAGE cut-outs were immersed in 90 µL of 100 mM sodium phosphate buffer solution with 0.15 M NaCl (42.6 mg $NaH_2PO_4 \cdot H_2O$, 93.7 mg Na_2HPO_4, 0.3 mL 0.5 M NaCl in 10 mL MilliQ water; pH adjusted to 7.5). One mg of the mass spec cleavable cross-linker disuccinimidyl sulfoxide (DSSO; Thermo Scientific A33545) was resuspended in 51.5 µL of dimethyl sulfoxide (DMSO) to reach a final concentration of 50 mM DSSO. 10 µL of 50 mM DSSO was then added to each sample yielding a final DSSO concentration of 5 mM per sample. Samples were briefly vortexed and then incubated at room temperature for 30 minutes. The cross-linking reaction was then stopped with the addition of 2 µL of 1 M Tris-HCl. Samples were briefly vortexed and incubated for 15 minutes at room temperature. The solution was then removed and replaced with MilliQ water. Samples were then stored at 4 °C until being processed for mass spectrometry analysis. While results from this method are convincing, in gel chemical cross-linking is a relatively new technique [22], and putative artifacts cannot be excluded due to the lack of a strong control.

Sample preparation of Tricine-SDS-PAGE and BN-PAGE gel cut-outs for mass spectrometry

The Coomassie-stained gel bands were destained with a mixture of acetonitrile (Chromasolv, Sigma-Aldrich) and 50 mM ammonium bicarbonate (Sigma-Aldrich). The proteins were reduced using 10 mM DTT (Roche) and alkylated with 50 mM iodoacetamide (IAA). Trypsin (Promega; Trypsin Gold, Mass Spectrometry Grade) digestion was carried out at 37 °C overnight in 50 mM ammonium bicarbonate.

Chymotrypsin (Roche) digestion was carried out at 25 °C for five hours in 50 mM ammonium bicarbonate. GluC (Roche) digestion was carried out at 37 °C overnight in 50 mM ammonium bicarbonate. Digestion was stopped with 10% formic acid (FA) and peptides were extracted twice with 5% FA for 10 min in a cooled ultrasonic bath. Extracted peptides were pooled and desalted using C18 Stagetips [23].

Liquid chromatography separation coupled to mass spectrometry

Peptides were analyzed on an UltiMate 3000 HPLC RSLC nanosystem (Thermo Fisher Scientific) coupled to a Q Exactive HF-X, equipped with a nano-spray ion source using coated emitter tips (PepSep, MSWil). Samples were loaded on a trap column (Thermo Fisher Scientific, PepMap C18, 5 mm × 300 μm ID, 5 μm particles, 100 Å pore size) at a flow rate of 25 μL min^{-1} using 0.1% TFA as the mobile phase. After 10 min, the trap column was switched in-line with the analytical C18 column (Thermo Fisher Scientific, PepMap C18, 500 mm × 75 μm ID, 2 μm, 100 Å) and peptides were eluted by applying a segmented linear gradient from 2% to 80% solvent B (80% acetonitrile, 0.1% formic acid; solvent A 0.1% formic acid) at a flow rate of 230 nL/min over 60 min. The mass spectrometer was operated in data-dependent mode, survey scans were obtained in a mass range of 350-1600 m/z with lock mass activated and at a resolution of 120 000 at 200 m/z and an AGC target value of 1e6. The 15 most intense ions were selected with an isolation width of 1.2 Thomson for a maximum of 150 ms, fragmented in the HCD cell at stepped normalized collision energy at 26%, 28%, and 30%. The spectra were recorded at an AGC target value of 1e5 and a resolution of 60 000. Peptides with a charge of +1, or > +7 were excluded from fragmentation. the peptide match feature was set to preferred, the exclude isotope feature was enabled, and selected precursors were dynamically excluded from repeated sampling for 20 seconds within a mass tolerance of 8 ppm.

Data analysis for identification of BN-PAGE and Tricine-SDS-PAGE bands

For peptide and protein identification, raw data were processed using the MaxQuant software package [24] (version 1.6.6.0) and spectra searched against *Nitrososphaera viennensis* or *Nitrosocaldus cavascurensis* reference proteomes (Uniprot, downloaded fall 2021), with a starting site modification (see below) to AmoC4 (Uniprot

accession: A0A060HLS1) in the *N. viennensis* proteome,and a database containing common contaminants. The search was performed with full trypsin specificity (or corresponding enzyme used in the digestion) and a maximum of two missed cleavages at a protein and peptide spectrum match false discovery rate of 1%. Carbamidomethylation of cysteine residues was set as a fixed modification and oxidation of methionine and N-terminal acetylation as variable modifications. The output option of iBAQ with log fit was selected. All other parameters were left at default. All proteomic data is deposited in the PRIDE database under accession numbers PXD035349, PXD034632, and PXD034475 for BN-PAGE of *N. viennensis*, BN-PAGE of *N. cavascurensis*, and cross-linked samples, respectively. [25].

Data analysis for identification of BN-PAGE cross-linked bands

Peptide and protein identification was performed as described as except with MaxQuant version 1.6.17.0 and with the a reference proteome that was not corrected for AmoC4. The raw files were converted per cv into raw files in FreeStyle.

To identify cross-linked peptides, the raw data were searched with either MS Annika [26] in Proteome Discoverer 2.3 or with MeroX 2.0 [27] against the sequences of the top abundant protein hits (with at least 10 MS/MS counts) from the MaxQuant search. Although it had less than 10 MS/MS counts, the protein encoded by NVIE_004550 (AmoZ) was also added based on other proteomic and syntenic analyses. DSSO was selected as the cross-linking chemistry. Carbamidomethyl on Cys was set as a fixed modification and oxidation of Met and protein N-terminal acetylation as variable modifications. Enzyme specificity was selected according to the protease used for digestion (trypsin). Search results were filtered for 1% FDR on the PSM level limiting the precursor mass deviation to 10 ppm. Further filtering was done for only non-decoy and high confidence PSMs in MS Annika and for a score higher than 50 in MeroX 2.0. The website tool http://crosslinkviewer.org/ was used to draw the XL maps.

Solvent accessible surface distances (SASD) between cross-linked residues were calculated and plotted on the AlphaFold *N. viennensis* structure model with Jwalk, and scored with the MNXL program to assess whether they violate distance criteria [28]. Cross-links were considered “matched” (or allowed) if the SASD between the cross-linked residues was < 35 Å (based on Cα-Cα distances).

Start site of amoC4 *in* N. viennensis

Six homologs of *amoC* (*amoC1-6*) are present in the genome of *N. viennensis*. Predicted subunit start sites for AmoC1-3, AmoC5, and AmoC6 are consistent across annotations (GenBank and RefSeq; Fig. S10). However, the start site of AmoC4 is significantly different between the two annotations. Additionally, there is a third possibility based on a methionine that would resemble the start site of all other AmoC homologs. Based on AmoC alignments in all AOA and transcriptional information from Reyes et al. (2020) [6], it was determined that the most accurate start site for this protein resides at the third option, resembling that of the other AmoC homologs in *N. viennensis* (Fig. S10). The proteome downloaded from Uniprot was manually annotated to reflect this decision. This is the proteome used for BN-PAGE and Tricine-SDS-PAGE reference files (but not for cross-linking analysis). With this correction, it is not possible to distinguish between AmoC4 and AmoC6 protein groups in any of the samples (i.e. finding unique peptides for both proteins). However, if the original proteome from Uniprot is used (downloaded Fall 2021), MaxQuant will identify unique peptides for both AmoC4 and AmoC6. Based on the current analysis, this is an inaccurate interpretation of the data. With the corrected AmoC4 annotation, only unique peptides from AmoC6 are found thus preventing the definitive separation of AmoC4 and AmoC6 into individual protein groups. Regardless, this suggests that most of the AmoC4/AmoC6 peptide signal is coming from AmoC6. To further verify that AmoC4 is not playing a significant role, the raw data from the chymotrypsin digest was subjected to an unspecific and semi-specific closed search for unique peptides from AmoC4. An open search was also carried out using the FragPipe software (version 17.1) [29]. None of these searches revealed unique AmoC4 peptides therefore strengthening the argument that the primary structural homolog in *N. viennensis* is AmoC6. Peptide coverage maps (Dataset S1) for the three digests (trypsin, GluC, and chymotrypsin) were created using Protein Coverage Summarizer (v1.3.8056) (https://github.com/PNNL-Comp-Mass-Spec/protein-coverage-summarizer/releases).

BN-PAGE correlation analysis

A Kendall correlation using R was used to find proteins that had a similar migration pattern with AmoABC. Any protein expected to be a part of the AMO complex should be in high abundance. Therefore, only the top 50% proteins, based on iBAQ abundance, were used for the analysis. In some cases, proteins with short sequences did not meet the requirement for iBAQ normalization (iBAQ is calculated by dividing intensity values by the number of peptides greater than six amino acids and less than 30 from an *in silico* digestion to normalize for length). This resulted in errors in the iBAQ calculation for short proteins with zero peptides meeting these requirements for the chosen digestive enzyme (in this case Trypsin/P as selected in MaxQuant). For these small proteins (NVIE_004140 and NVIE_022250 in *N. viennensis*; NCAV_0491 in *N. cavascurensis*), the unnormalized output intensity was used. This would be the same as dividing the intensity by one (representing one *in silico* peptide between six and 30 amino acids in length). As these are small proteins, this should produce an iBAQ value that reflects an appropriate size normalization when compared to other proteins. Each protein was correlated with every other protein using the function cor.test in R with method="kendall" and use="complete.obs" using iBAQ values. The calculated p values were adjusted using the Benjamini-Hochberg method. Results were filtered for proteins correlated with one of the AMO proteins (AmoA, AmoB, or AmoC), a correlation (tau) greater than or equal to 0.7, and an adjusted p value lower than or equal to 0.001.

In *N. viennensis*, highly identical proteins (>90% identity, BLASTp [4, 5]) to AmoC and AmoX were excluded from the correlation analysis. This included proteins groups AmoC1/C2, AmoC3, and NVIE_022250. Protein groups AmoC1/C2 and NVIE_022250 followed the same profile as other AMO subunits, but were excluded as they are considered to be homologs that are far less abundant in terms of both protein and transcript abundance and are therefore not representing the primary components of the AMO complex under these growth conditions. However, it is possible that a small proportion of AMO subunits contain these homologs. R scripts can be found here: https://github.com/hodgskiss/Archaeal_AMO.

For genes of interest, conservation across AOA was checked according to the data set curated in Abby et al. (2020) [30]. A gene was considered exclusive to AOA if it was found in AOA but not found in any species outside of known AOA. The dataset from this study was provided by the authors and can be found here: https://github.com/hodgskiss/Archaeal_AMO.

Transcriptomic clustering

Transcriptomic reads from Reyes et al. (2020) [6] were re-processed taking into account strandedness and using hisat2 (version 2.1.0) [31] rather than bowtie2 for read mapping. This reanalysis greatly changed the number of reads assigned to *amoC* genes as it only accounted for reads that could be uniquely mapped to each gene or homolog. This particularly changed the abundance for *amoC4*. Count values were evaluated using featureCounts (v2.0.0) [32]. Counts were then normalized using the transcripts per kilobase million (TPM) method. TPM is calculated by dividing each gene count by the total length of the gene in kilobases giving a reads per kilobase value. This "reads per kilobase" value was summed up for all genes in a sample and divided by 1 000 000 to give a scaling factor. Each genes' "reads per kilobase" was divided by this scaling factor to give the final TPM. TPM values were converted to $\log_2$ values and clustered using hierarchal clustering with the default values of heatmap.2 [33] in R version 3.6.3 [34]. Genes were split into 15 clusters. Three clusters represented the genes with the highest abundance of transcripts in both the copper replete and copper limited cultures. Clustered data is visualized using Anvio 7.1 [35] (Fig. S2).

Structural search for missing AMO sections in archaea

Extended pieces of bacterial AmoB and AmoC were trimmed and collected from alignments of combined archaea and bacteria species using trimal (v1.4rev15) [36]. One sequence was removed from AmoB sequences as it was not actually the AmoB subunit. A second was removed due to the inclusion of stop codons in the sequence. Trimmed sections were used to create HMMs and then searched against archaeal species to look for proteins that might supply these missing pieces. No hits were found. Therefore, a

structural search for proteins containing transmembrane helices in *N. viennensis* was carried out for highly transcribed genes.

TPM reads from Reyes et al. 2020 [6] were averaged for the five replete conditions and sorted by highest abundance to obtain the top 100 transcribed genes. The GenBank translated CDS region for *N. viennensis* was analyzed using Phobius [37] to identify transmembrane helices and signal peptides in all proteins. The highest transcribed genes were then filtered to include genes with 1-3 predicted transmembrane helices. Candidate genes were then analyzed for conservation in AOA and correlation in BN-PAGE gels from *N. viennensis*.

Predicted model of archaeal AMO using AlphaFold-multimer

Sequences for AmoA, AmoB, AmoC, AmoX, AmoY (NVIE_004540), and AmoZ (NVIE_004550) from *N. viennensis* were used for AlphaFold2.1 [38–40] predictions. In the case of AmoB and AmoZ , predicted signal peptides were removed based on predictions using SignalP 5.0 (archaea) [41]. AmoC6 was used to represent the AmoC subunit. For *N. cavascurensis*, sequences for AmoA, AmoB, AmoC, AmoX (NCAV_0491) , AmoY (NCAV_0488), and AmoZ (NCAV_0486) were used after predicted signal peptides were removed based on predictions using SignalP 5.0 (archaea) [41]. The top ranked model for *N. viennensis* and *N. cavascurensis* was used for images and analysis. Rankings were based on per-residue confidence (pLDDT) scores. All images were generated in PyMOL [42].

Identification and alignments of AmoXYZ in ammonia oxidizing archaea

Amino acid sequences for AmoX, AmoY, and AmoZ were obtained from the genes *amoX*, NVIE_004540, and NVIE_004550, respectively. Homologs in other AOA were initially searched for using blastp (v2.12.0+) [12] with a threshold of 1e-4. Not all species had identified hits. A more sensitive analysis was performed by creating an HMM model using hmmbuild (HMMER 3.3, hmmer.org) for each new subunit from the top BLASTp hit from each species with a BLASTp result. The HMMs for AmoX, AmoY, and AmoZ were then used with hmmsearch (HMMER 3.3, hmmer.org) in all collected AOA genomes. This produced hits in all species for all subunits except AmoY and AmoZ

in Thaumarchaeota archaeon J079, a MAG that is also missing AmoB and is only 84% complete [43], and AmoZ in the GenBank protein file for "*Ca.* Cenarchaeum symbiosum A". A reanalysis of the "*Ca.* Cenarchaeum symbiosum A" genome using prodigal was able to identify a coding sequence for AmoZ while maintaining the coding sequences for all other AMO subunits. A complete list of all identified AMO subunits in the collected species, as well as protein alignments for all six AMO subunits, can be found at https://github.com/hodgskiss/Archaeal_AMO .

634 **Supplementary Figures**

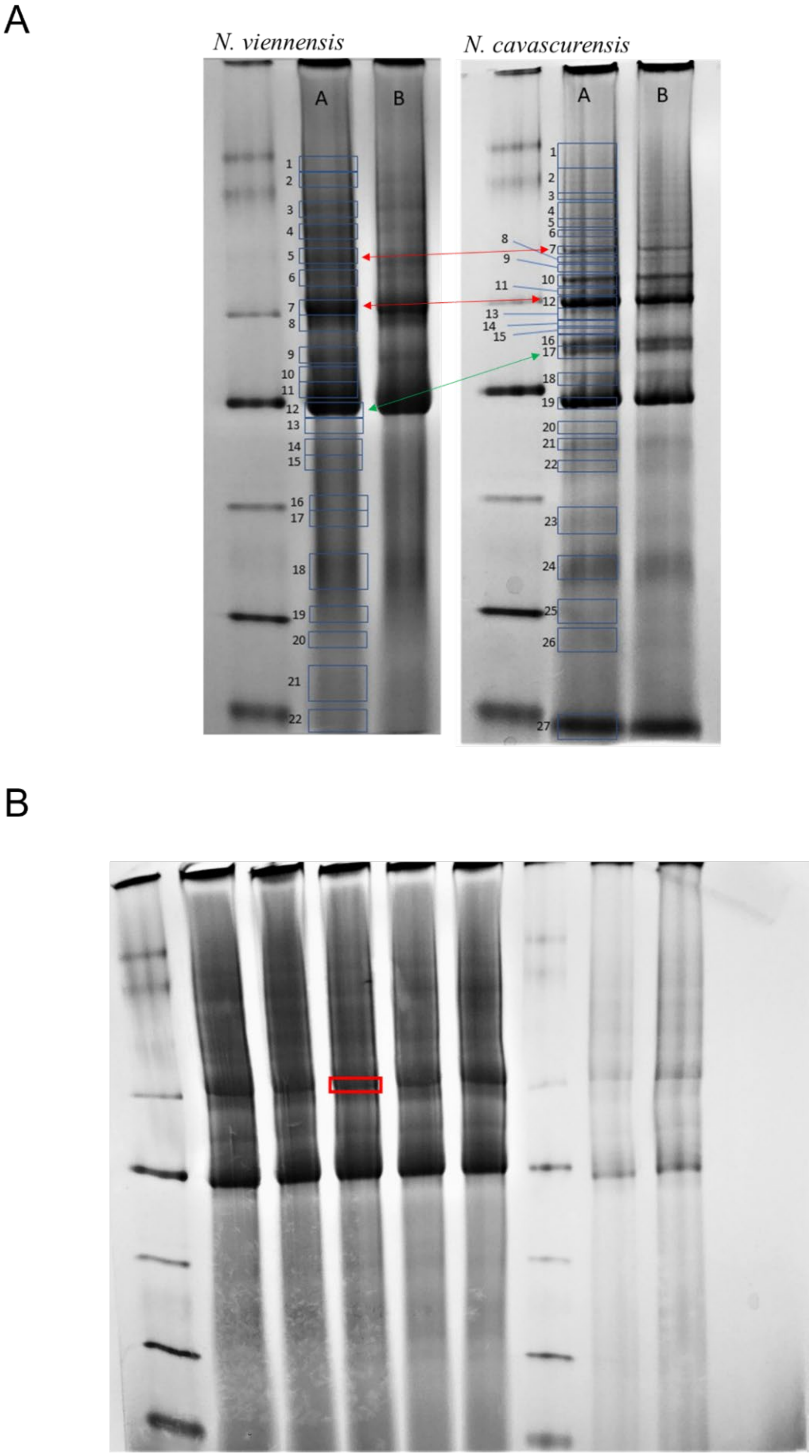

635

Figure S1. BN-PAGE gels of *N. viennensis* and *N. cavascurensis*. A) Comparison of protein membrane fractions between two species on BN-PAGE gels. Ladder and gels are the same. Blue boxes represent cut bands for proteomic analysis. Red arrows indicate bands of high AMO content that correspond between the two species. Green arrow points to a third AMO peak in both species that is found at different heights. Lanes A and B within **(A)** represent identical gel and sample conditions in each gel. **B)** BN-PAGE gel of *N. viennensis* used for in-gel cross-linking. The red box indicates the cut-out used for in-gel cross-linking with DSSO and subsequent mass spectrometry analysis. This cut-out corresponds to band 7 within Figure 1A..

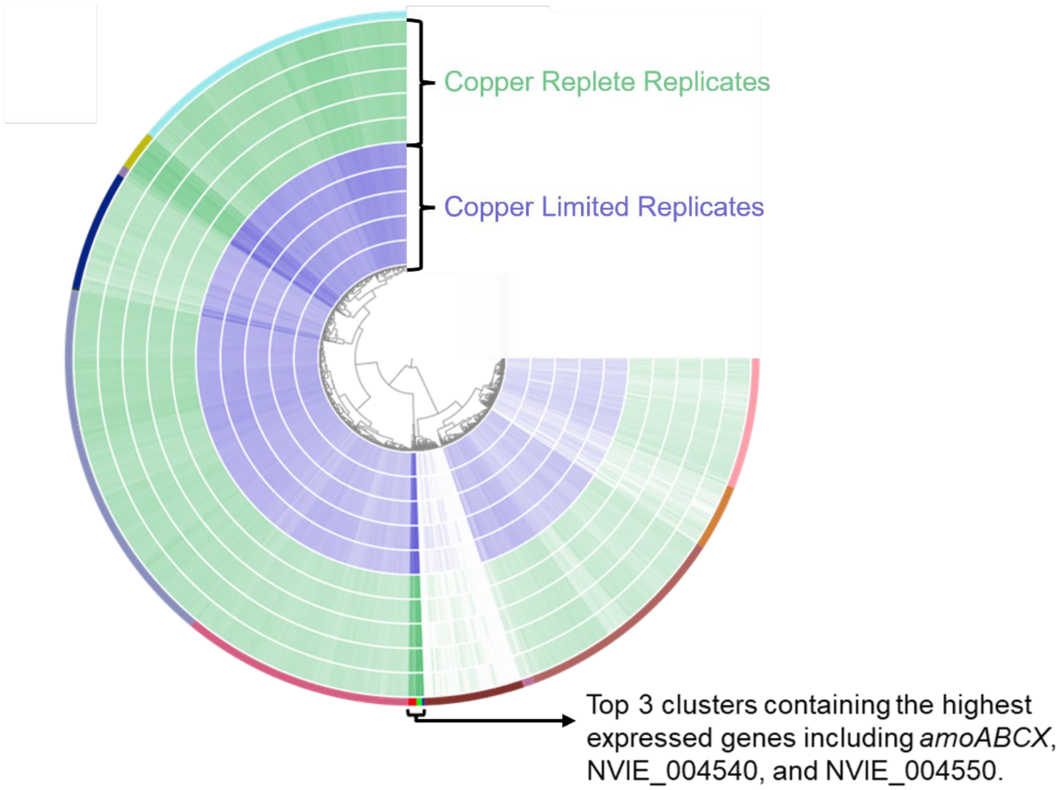

Figure S2. Transcriptomic clustering analysis of *amo* genes in *N. viennensis*. Clustering analysis of gene expression in *N. viennensis* under copper replete and limited conditions from Reyes et al. (2020) [6]. Genes were clustered into 15 separate clusters represented by different colors on the outer edge of the circular plot.

PmoA/AmoA

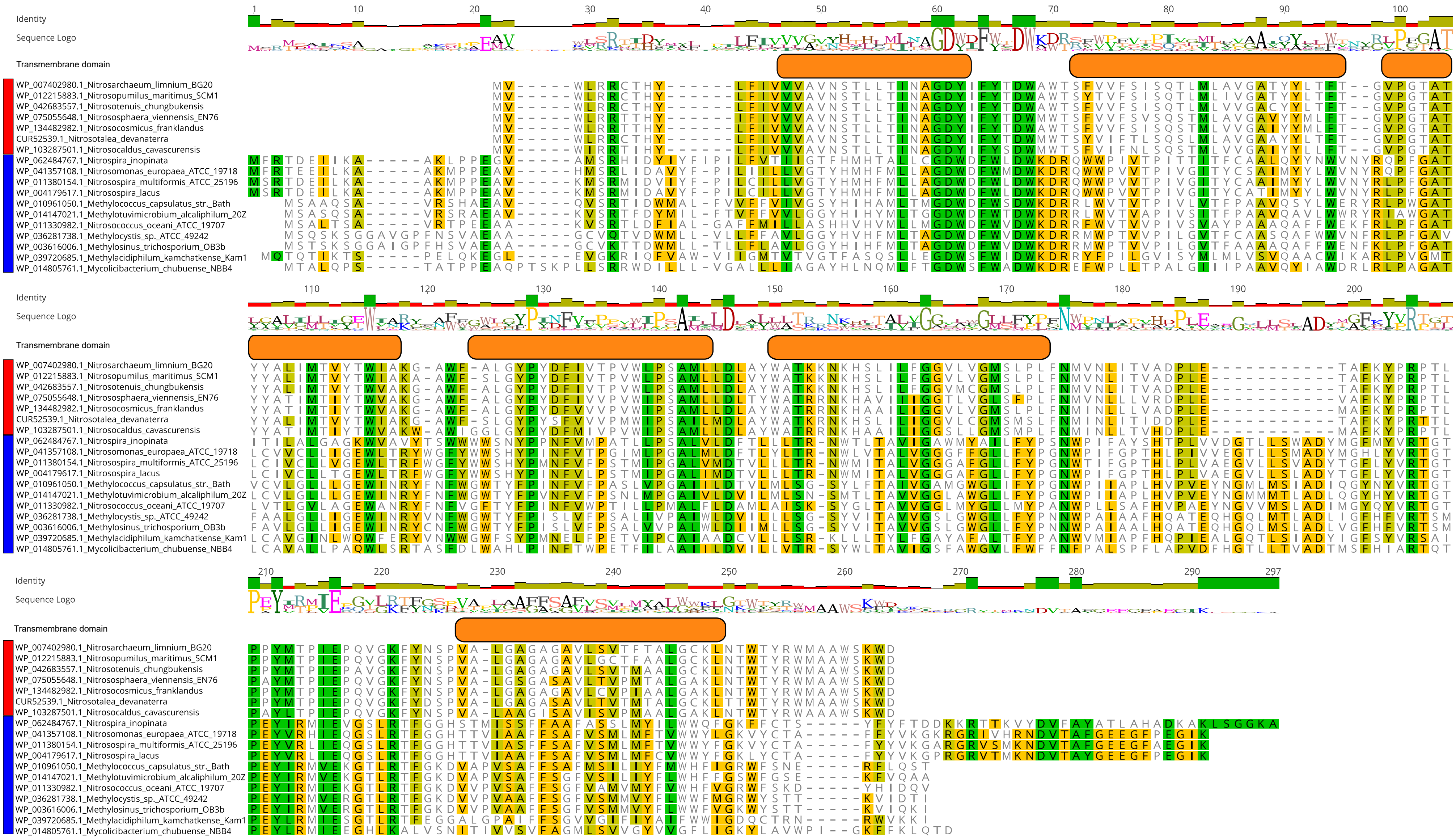
Identity
Sequence Logo
Transmembrane domain
WP_007402980.1_Nitrosarchaeum_limnium_BG20
WP_012215883.1_Nitrosopumilus_maritimus_SCM1
WP_042683557.1_Nitrosotenuis_chungbukensis
WP_075055648.1_Nitrososphaera_viennensis_EN76
WP_134482982.1_Nitrosocosmicus_franklandus
CUR52539.1_Nitrosotalea_devanaterra
WP_103287501.1_Nitrosocaldus_cavascurensis
WP_062484767.1_Nitrospira_inopinata
WP_041357108.1_Nitrosomonas_europaea_ATCC_19718
WP_011380154.1_Nitrosospira_multiformis_ATCC_25196
WP_004179617.1_Nitrosospira_lacus
WP_010961050.1_Methylococcus_capsulatus_str._Bath
WP_014147021.1_Methylotuvimicrobium_alcaliphilum_20Z
WP_011330982.1_Nitrosococcus_oceani_ATCC_19707
WP_036281738.1_Methylocystis_sp._ATCC_49242
WP_003616006.1_Methylosinus_trichosporium_OB3b
WP_039720685.1_Methylacidiphilum_kamchatkense_Kam1
WP_014805761.1_Mycolicibacterium_chubuense_NBB4

Figure S3. Protein alignments of PmoA/AmoA subunits from selected species. Percent identity of amino acids across species are indicated with bar chart along the top. Sequence logo visually represents the proportion of amino acids at each position. Orange bars represent transmembrane helices as found in the crystal structure of PMO from *Methylosinus trichosporium* OB3b [44]. Red bars represent archaeal species and blue bars represent bacterial species.

PmoB/AmoB

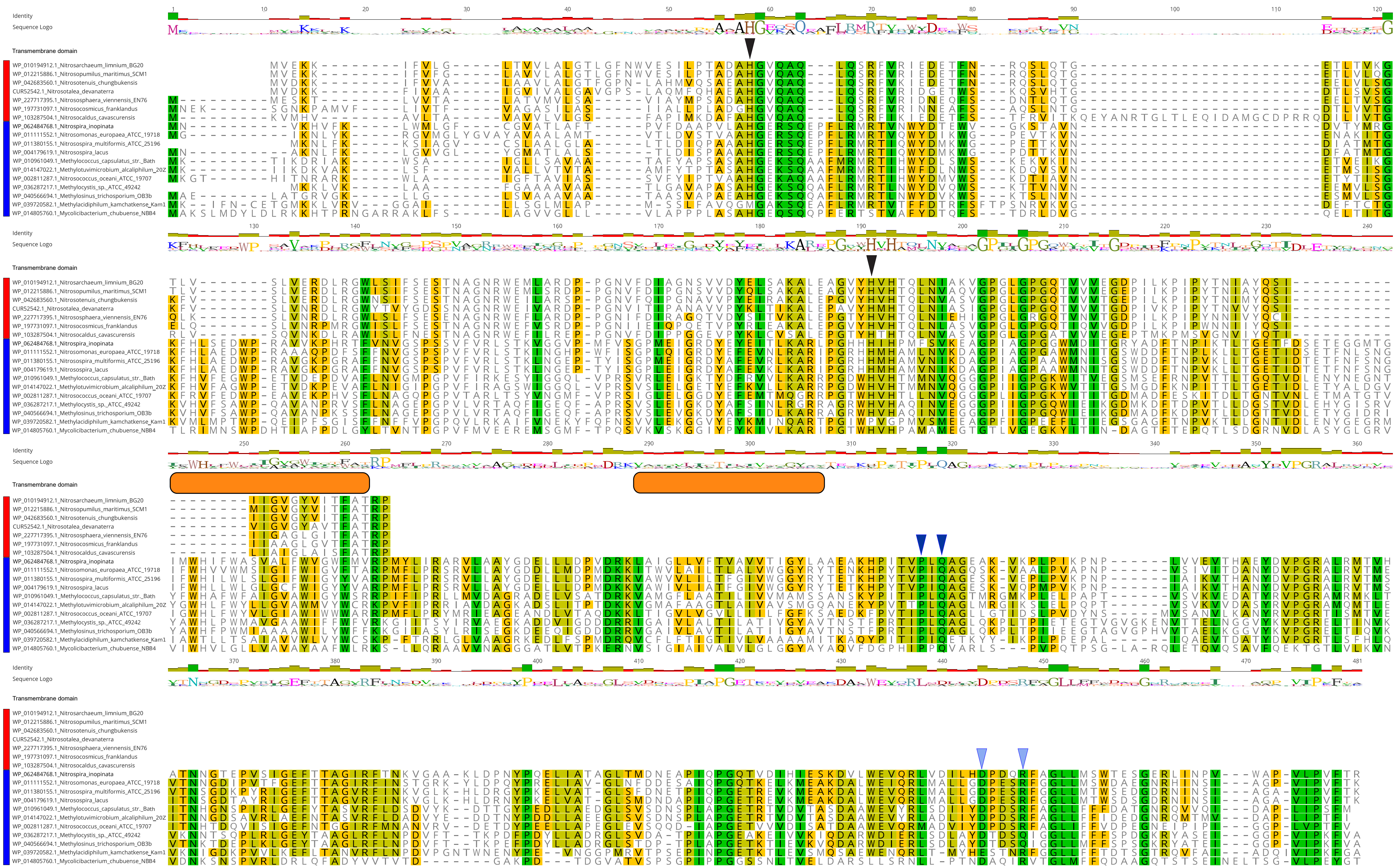
Identity
Sequence Logo
Transmembrane domain
WP_010194912.1_Nitrosarchaeum_limnium_BG20
WP_012215886.1_Nitrosopumilus_maritimus_SCM1
WP_042683560.1_Nitrosotenuis_chungbukensis
CUR52542.1_Nitrosotalea_devanaterra
WP_227717395.1_Nitrososphaera_viennensis_EN76
WP_197731097.1_Nitrosocosmicus_franklandus
WP_103287504.1_Nitrosocaldus_cavascurensis
WP_062484768.1_Nitrospira_inopinata
WP_011111552.1_Nitrosomonas_europaea_ATCC_19718
WP_011380155.1_Nitrosospira_multiformis_ATCC_25196
WP_004179619.1_Nitrospira_lacus
WP_010961049.1_Methylococcus_capsulatus_str._Bath
WP_014147022.1_Methylotuvimicrobium_alcaliphilum_20Z
WP_002811287.1_Nitrosococcus_oceani_ATCC_19707
WP_036287217.1_Methylocystis_sp._ATCC_49242
WP_040566694.1_Methylosinus_trichosporium_OB3b
WP_039720582.1_Methylacidiphilum_kamchatkense_Kam1
WP_014805760.1_Mycolicibacterium_chubuense_NBB4

Figure S4. Protein alignments of PmoB/AmoB subunits from selected species. Percent identity of amino acids across species is indicated with bar chart along the top. Sequence logo visually represents the proportion of amino acids at each position. Orange bars represent transmembrane helices as found in the crystal structure of PMO from *Methylosinus trichosporium* OB3b [44]. Red bars represent archaeal species and blue bars represent bacterial species. Conserved metal binding residues (with the exception of *Methylacidphilum kamchatkense* Kam1 from Verrucomicrobia) are marked with black arrowsr. Blue arrows indicate conserved amino acids that could be facilitating protomer interactions based on close proximity in the cryo-EM structure of *Methylococcous capsulatus* str. Bath (PDB structure: 7S4H) [7]. Light blue and dark blue arrows represent PmoB/AmoB residues from different protomers.

PmoC/AmoC

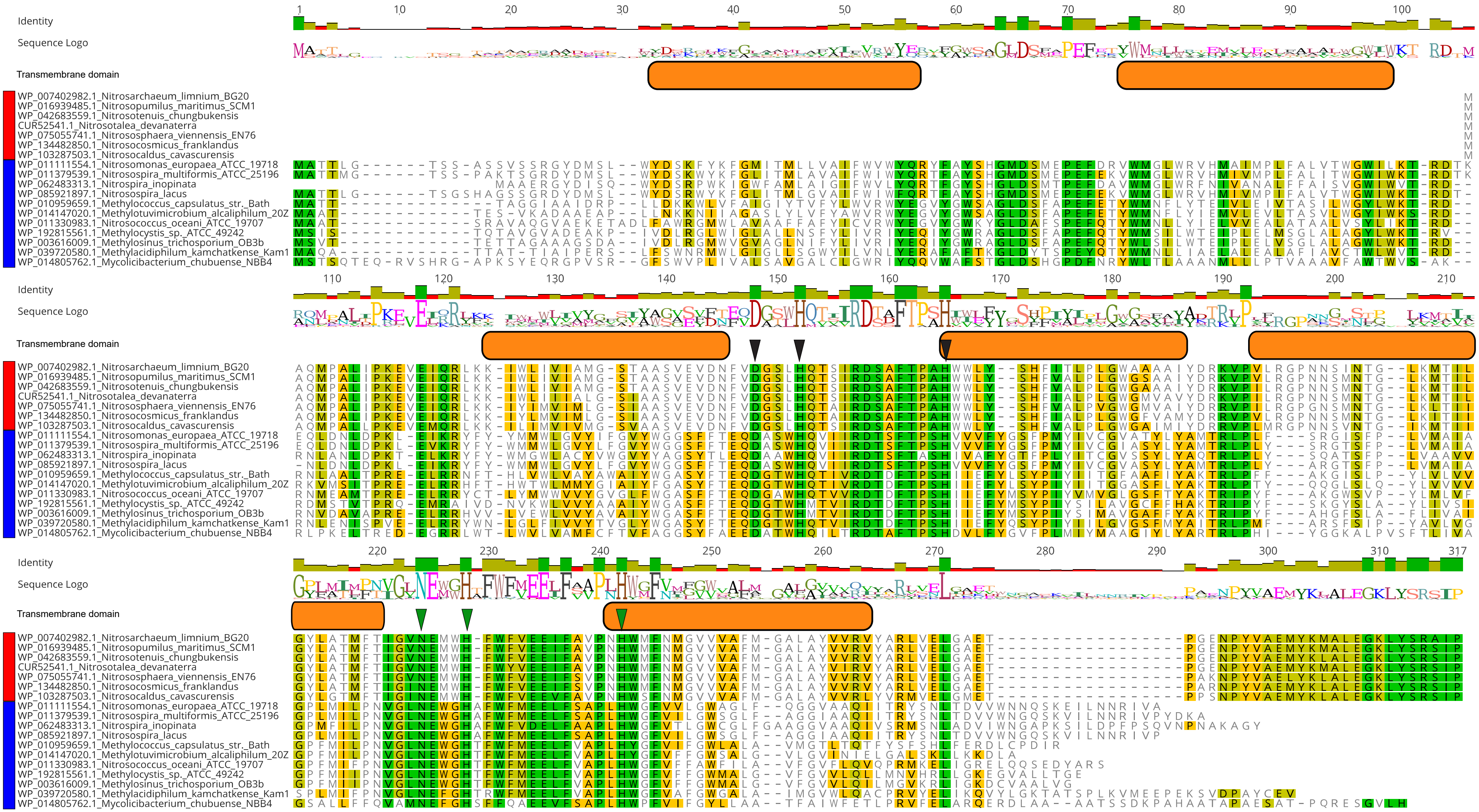
Identity
Sequence Logo
Transmembrane domain
WP_007402982.1_Nitrosarchaeum_limnium_BG20
WP_016939485.1_Nitrosopumilus_maritimus_SCM1
WP_042683559.1_Nitrosotenuis_chungbukensis
CUR52541.1_Nitrosotalea_devanaterra
WP_075055741.1_Nitrososphaera_viennensis_EN76
WP_134482850.1_Nitrosocosmicus_franklandus
WP_103287503.1_Nitrosocaldus_cavascurensis
WP_011111554.1_Nitrosomonas_europaea_ATCC_19718
WP_011379539.1_Nitrosospira_multiformis_ATCC_25196
WP_062483313.1_Nitrospira_inopinata
WP_085921897.1_Nitrosospira_lacus
WP_010959659.1_Methylococcus_capsulatus_str._Bath
WP_014147020.1_Methylotuvimicrobium_alcaliphilum_20Z
WP_011330983.1_Nitrosococcus_oceani_ATCC_19707
WP_192815561.1_Methylocystis_sp._ATCC_49242
WP_003616009.1_Methylosinus_trichosporium_OB3b
WP_039720580.1_Methylacidiphilum_kamchatkense_Kam1
WP_014805762.1_Mycolicibacterium_chubuense_NBB4

Figure S5. Protein alignments of PmoC/AmoC subunits from selected species. Percent identities of amino acids across species is indicated with bar chart along the top. Sequence logo visually represents the proportion of amino acids at each position. Orange bars represent transmembrane helices as found in the crystal structure of PMO from *Methylosinus trichosporium* OB3b [44]. Red bars represent archaeal species and blue bars represent bacterial species. Conserved metal binding residues are marked with black arrows. Conserved metal binding residues for a newly proposed metal site [7] are marked with green arrows.

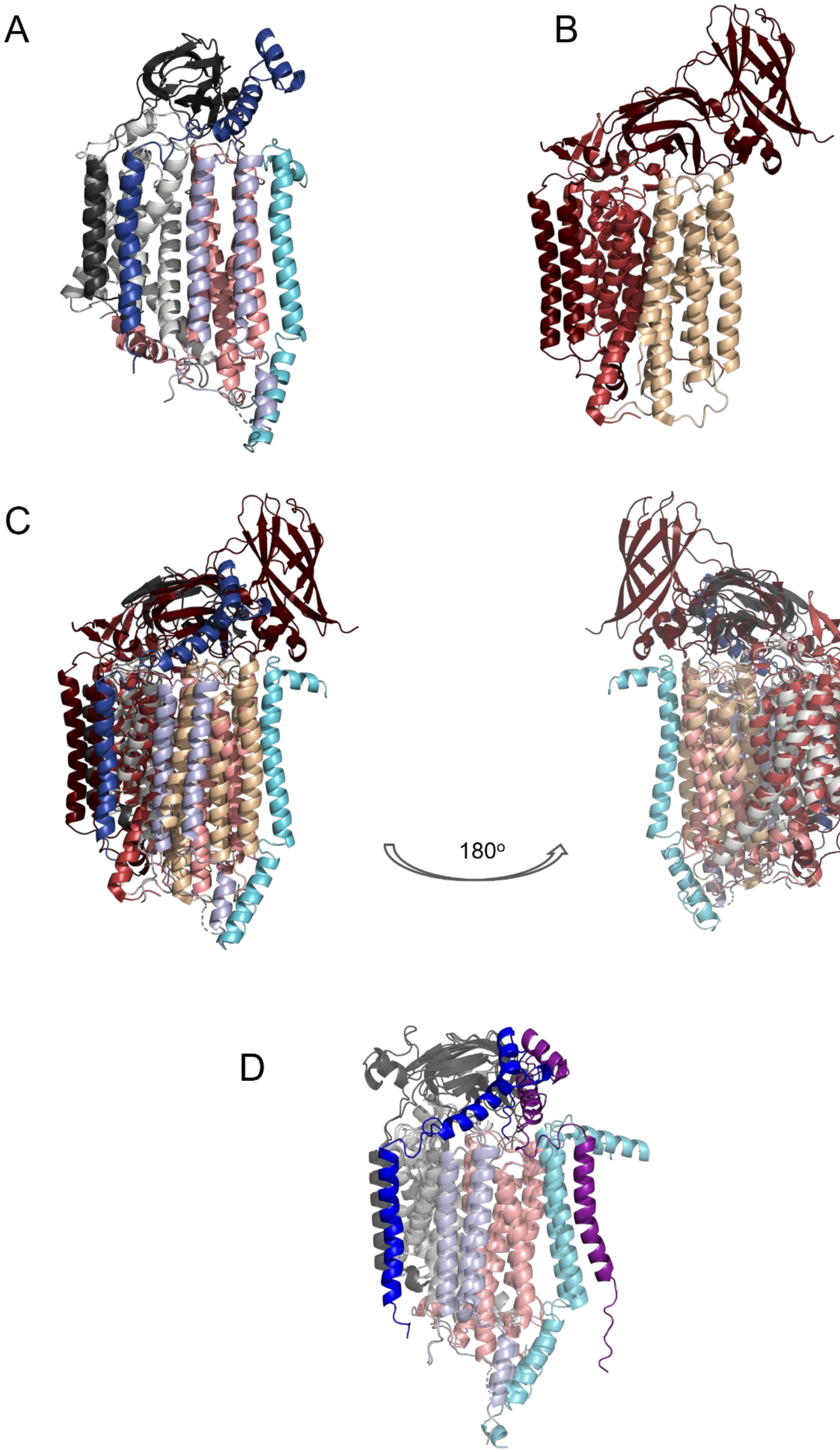
A
B
C
180°
D

Figure S6. Structural comparison between archaeal AMO and bacterial PMO. A) AlphaFold model of *N. viennenis*. **B)** Cryo-EM structure of the particulate methane monooxygenase (PDB:7S4H, pMMO) from *Methylococcus capsulatus* (Bath) [45] downloaded from the Protein Data Bank (PDB) [46]. **C)** Superimposition of the AlphaFold model of *N. viennensis* **(A)** on the crystal structure of the particulate methane monooxygenase (PDB:7S4H, pMMO) from *Methylococcus capsulatus* (Bath) **(B)**, with an RMSD 3.382 Å (PyMOL command "cealign"). Subunits are colored as follows: AmoA, light grey; AmoB, black; AmoC, salmon; AmoX, lavender; AmoY, cyan; AmoZ, blue; PmoA, dark salmon; PmoB, dark red; PmoC, tan. **D)** Superimposition of the AlphaFold model of *N. cavascurensis* on the AlphaFold model of *N. viennensis* (PyMOL command "align") with an RMSD of 1.67Å. To highlight substantial differences, all subunits are colored the same and are faded except for AmoZ: *N. viennensis* AmoZ is colored bright blue and *N. cavascurensis* is colored purple. Images generated using PyMOL [42].

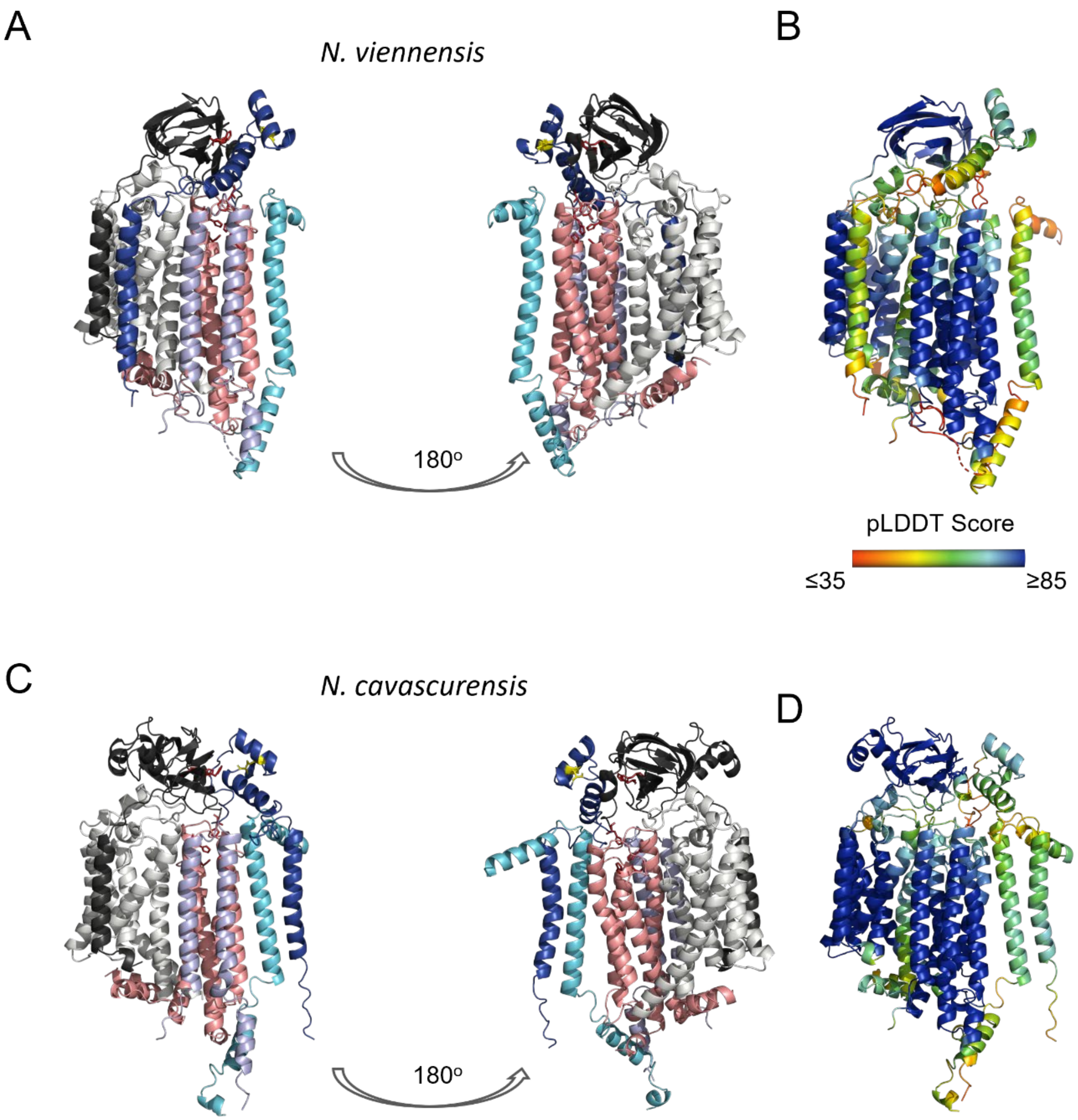

Figure S7. AlphaFold models and pLDDT confidence scores of *N. viennensis* and *N. cavascurensis* heterohexamers. A,C) *N. viennensis* hexamer **(A)** and *N. cavascurensis* hexamer **(C)** in cartoon representations, revealing the position of the Cu_B and Cu_C copper centers in magenta sticks, and the disulfide bonds in yellow. Subunits are colored as follows: AmoA, light grey; AmoB, black; AmoC, salmon; AmoX, lavender; AmoY, cyan; AmoZ, blue. **B, D)** The *N. viennensis* **(B)** and *N. cavascurensis* **(D)** models colored according to the per residue pLDDT confidence scores from red (≤ 35) to blue (≤ 100). 57% of the residues in *N. viennensis* model and 52% in *N. cavascurensis* model were modeled with a relatively high accuracy of > 70%.

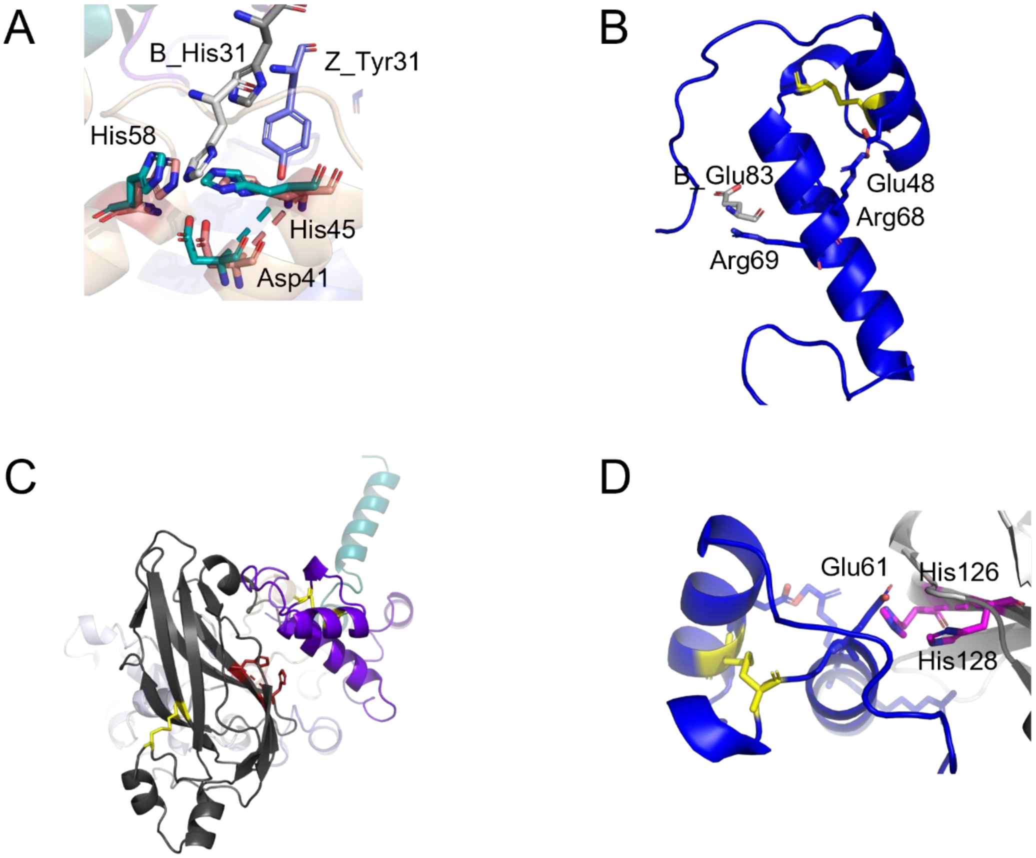

Figure S8. Features of the AlphaFold models of *N. viennensis* and *N. cavascurensis* heterohexamers. A) Closeup view of the residues involved in the coordination of the CuC copper center. Canonical residues His58, His45, Asp41 from AmoC (*N.viennensis* numbering) are colored in salmon for the *N. viennensis* model and teal for the *N. cavascurensis* model, respectively. The conserved His31 from the N-terminus of AmoB which might be participating in the coordination sphere is depicted in white and grey for *N. viennensis* and *N. cavascurensis,* respectively. The conserved Tyr31 from AmoZ in *N. viennensis* is also located in the vicinity (4.5-7.2 Å) of the metal coordination sphere is depicted in blue. **B)** Closeup view of the residues involved in putatively stabilizing intra- and inter-subunit interactions between AmoZ (blue) and AmoB (grey) in *N. viennensis*. A hydrogen bond between conserved residues Glu48 and Arg68 could stabilize the extracellular helices of AmoZ in addition to the predicted disulfide bond (in yellow), while conserved Arg69 could form a hydrogen bond with Glu83 from AmoB.**C)** Top view of the extracellular domain in the *N. cavascurensis* model, illustrating the relative orientation of AmoB (grey) and AmoZ (purple). Note the disulfide bonds (in yellow) tethering the extended AmoB loop to the β-barrel, and between the AmoZ extracellular helices. The CuB center is depicted in dark red sticks.**D)** Closeup view of the residues involved in the coordination of the *N. viennensis* CuB copper center. Canonical residues His126 and His128 (AmoB) are depicted in magenta, while Glu61 from AmoZ located in the close vicinity of the center is in blue.

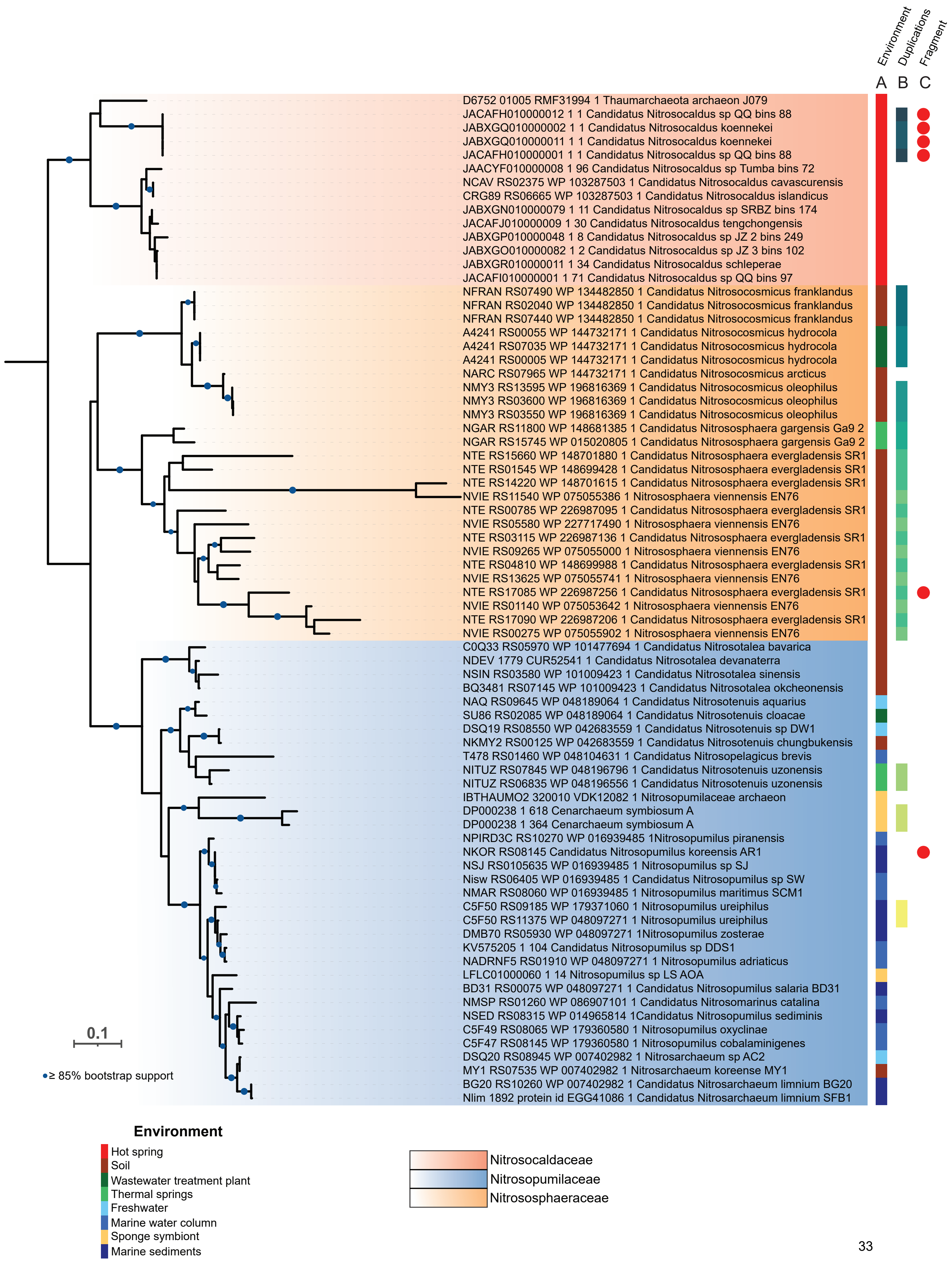

Environment
Duplications
Fragment
A
B
C
D6752 01005 RMF31994 1 Thaumarchaeota archaeon J079
JACAFH010000012 1 1 Candidatus Nitrosocaldus sp QQ bins 88
JABXGQ010000002 1 1 Candidatus Nitrosocaldus koennekei
JABXGQ010000011 1 1 Candidatus Nitrosocaldus koennekei
JACAFH010000001 1 1 Candidatus Nitrosocaldus sp QQ bins 88
JAACYF010000008 1 96 Candidatus Nitrosocaldus sp Tumba bins 72
NCAV RS02375 WP 103287503 1 Candidatus Nitrosocaldus cavascurensis
CRG89 RS06665 WP 103287503 1 Candidatus Nitrosocaldus islandicus
JABXGN010000079 1 11 Candidatus Nitrosocaldus sp SRBZ bins 174
JACAFJ010000009 1 30 Candidatus Nitrosocaldus tengchongensis
JABXGP010000048 1 8 Candidatus Nitrosocaldus sp JZ 2 bins 249
JABXGO010000082 1 2 Candidatus Nitrosocaldus sp JZ 3 bins 102
JABXGR010000011 1 34 Candidatus Nitrosocaldus schleperae
JACAFI010000001 1 71 Candidatus Nitrosocaldus sp QQ bins 97
NFRAN RS07490 WP 134482850 1 Candidatus Nitrosocosmicus franklandus
NFRAN RS02040 WP 134482850 1 Candidatus Nitrosocosmicus franklandus
NFRAN RS07440 WP 134482850 1 Candidatus Nitrosocosmicus franklandus
A4241 RS00055 WP 144732171 1 Candidatus Nitrosocosmicus hydrocola
A4241 RS07035 WP 144732171 1 Candidatus Nitrosocosmicus hydrocola
A4241 RS00005 WP 144732171 1 Candidatus Nitrosocosmicus hydrocola
NARC RS07965 WP 144732171 1 Candidatus Nitrosocosmicus arcticus
NMY3 RS13595 WP 196816369 1 Candidatus Nitrosocosmicus oleophilus
NMY3 RS03600 WP 196816369 1 Candidatus Nitrosocosmicus oleophilus
NMY3 RS03550 WP 196816369 1 Candidatus Nitrosocosmicus oleophilus
NGAR RS11800 WP 148681385 1 Candidatus Nitrososphaera gargensis Ga9 2
NGAR RS15745 WP 015020805 1 Candidatus Nitrososphaera gargensis Ga9 2
NTE RS15660 WP 148701880 1 Candidatus Nitrososphaera evergladensis SR1
NTE RS01545 WP 148699428 1 Candidatus Nitrososphaera evergladensis SR1
NTE RS14220 WP 148701615 1 Candidatus Nitrososphaera evergladensis SR1
NVIE RS11540 WP 075055386 1 Nitrososphaera viennensis EN76
NTE RS00785 WP 226987095 1 Candidatus Nitrososphaera evergladensis SR1
NVIE RS05580 WP 227717490 1 Nitrososphaera viennensis EN76
NTE RS03115 WP 226987136 1 Candidatus Nitrososphaera evergladensis SR1
NVIE RS09265 WP 075055000 1 Nitrososphaera viennensis EN76
NTE RS04810 WP 148699988 1 Candidatus Nitrososphaera evergladensis SR1
NVIE RS13625 WP 075055741 1 Nitrososphaera viennensis EN76
NTE RS17085 WP 226987256 1 Candidatus Nitrososphaera evergladensis SR1
NVIE RS01140 WP 075053642 1 Nitrososphaera viennensis EN76
NTE RS17090 WP 226987206 1 Candidatus Nitrososphaera evergladensis SR1
NVIE RS00275 WP 075055902 1 Nitrososphaera viennensis EN76
C0Q33 RS05970 WP 101477694 1 Candidatus Nitrosotalea bavarica
NDEV 1779 CUR52541 1 Candidatus Nitrosotalea devanaterra
NSIN RS03580 WP 101009423 1 Candidatus Nitrosotalea sinensis
BQ3481 RS07145 WP 101009423 1 Candidatus Nitrosotalea okcheonensis
NAQ RS09645 WP 048189064 1 Candidatus Nitrosotenuis aquarius
SU86 RS02085 WP 048189064 1 Candidatus Nitrosotenuis cloacae
DSQ19 RS08550 WP 042683559 1 Candidatus Nitrosotenuis sp DW1
NKMY2 RS00125 WP 042683559 1 Candidatus Nitrosotenuis chungbukensis
T478 RS01460 WP 048104631 1 Candidatus Nitrosopelagicus brevis
NITUZ RS07845 WP 048196796 1 Candidatus Nitrosotenuis uzonensis
NITUZ RS06835 WP 048196556 1 Candidatus Nitrosotenuis uzonensis
IBTHAUMO2 320010 VDK12082 1 Nitrosopumilaceae archaeon
DP000238 1 618 Cenarchaeum symbiosum A
DP000238 1 364 Cenarchaeum symbiosum A
NPIRD3C RS10270 WP 016939485 1Nitrosopumilus piranensis
NKOR RS08145 Candidatus Nitrosopumilus koreensis AR1
NSJ RS0105635 WP 016939485 1 Nitrosopumilus sp SJ
Nisw RS06405 WP 016939485 1 Candidatus Nitrosopumilus sp SW
NMAR RS08060 WP 016939485 1 Nitrosopumilus maritimus SCM1
C5F50 RS09185 WP 179371060 1 Nitrosopumilus ureiphilus
C5F50 RS11375 WP 048097271 1 Nitrosopumilus ureiphilus
DMB70 RS05930 WP 048097271 1Nitrosopumilus zosterae
KV575205 1 104 Candidatus Nitrosopumilus sp DDS1
NADRNF5 RS01910 WP 048097271 1 Nitrosopumilus adriaticus
LFLC01000060 1 14 Nitrosopumilus sp LS AOA
BD31 RS00075 WP 048097271 1 Candidatus Nitrosopumilus salaria BD31
NMSP RS01260 WP 086907101 1 Candidatus Nitrosomarinus catalina
NSED RS08315 WP 014965814 1Candidatus Nitrosopumilus sediminis
C5F49 RS08065 WP 179360580 1 Nitrosopumilus oxyclinae
C5F47 RS08145 WP 179360580 1 Nitrosopumilus cobalaminigenes
DSQ20 RS08945 WP 007402982 1 Nitrosarchaeum sp AC2
MY1 RS07535 WP 007402982 1 Nitrosarchaeum koreense MY1
BG20 RS10260 WP 007402982 1 Candidatus Nitrosarchaeum limnium BG20
Nlim 1892 protein id EGG41086 1 Candidatus Nitrosarchaeum limnium SFB1
0.1
≥ 85% bootstrap support
Environment
Hot spring
Soil
Wastewater treatment plant
Thermal springs
Freshwater
Marine water column
Sponge symbiont
Marine sediments
Nitrosocaldaceae
Nitrosopumilaceae
Nitrososphaeraceae

Figure S9. Phylogenetic tree of *amoC* gene nucleotide sequences in ammonia oxidizing archaea. Labels are colored according to GTDB[47] families: *Nitrosocaldaceae*-red, *Nitrosopumilaceae*-blue, *Nitrososphaeraceae*-orange. Blue circles represent ultrafast bootstrap values ≥ 85 %. Labels include the gene locus tag followed by the NCBI protein accession and species name. Added information next to the phylogenetic tree includes: (A) environment; (B) *amoC* duplications represented by different colors for each species; (C) *amoC* fragment sequences represented by a full circle.

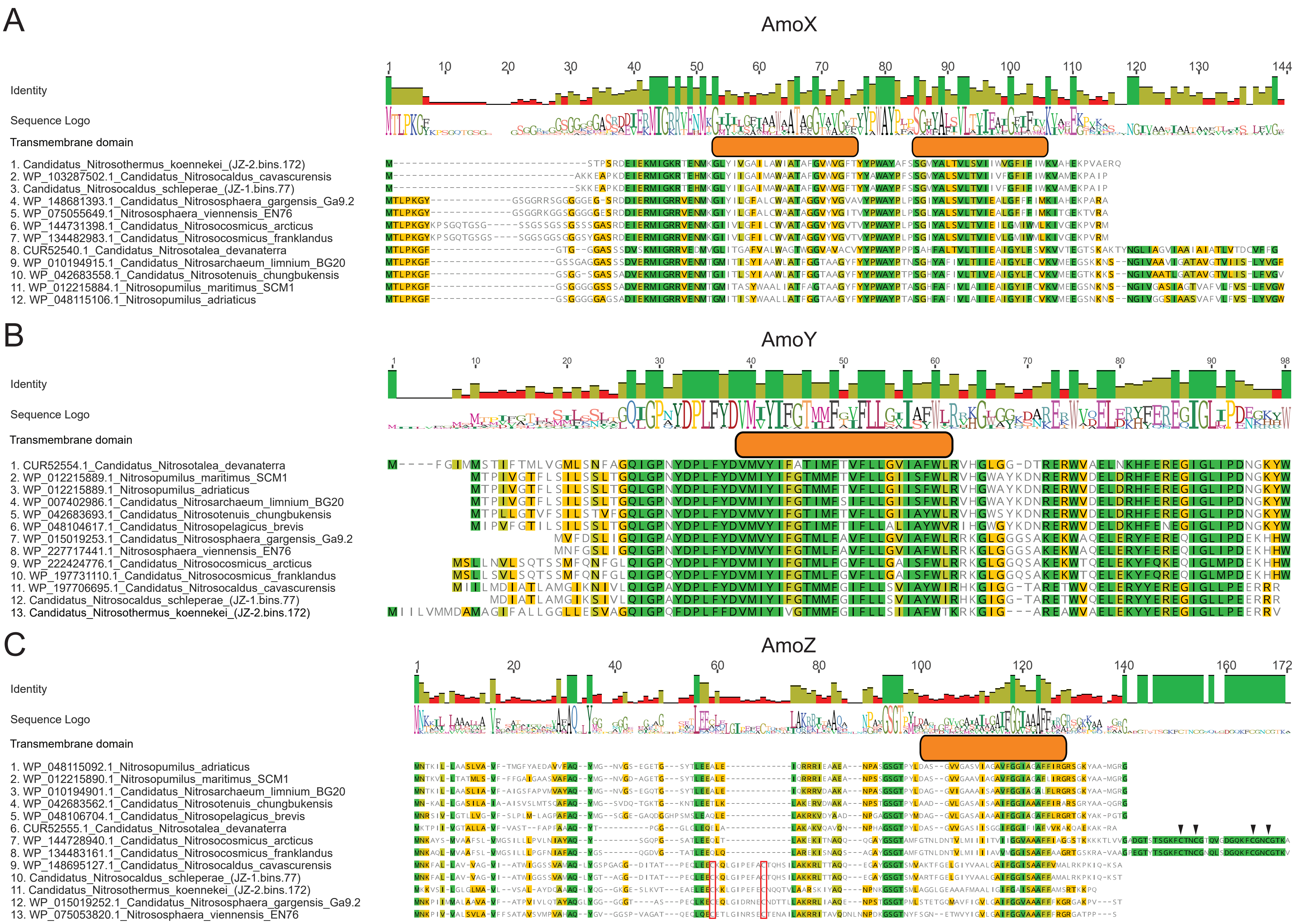
A
AmoX
Identity
Sequence Logo
Transmembrane domain
1. Candidatus_Nitrosothermus_koennekei_(JZ-2.bins.172)
2. WP_103287502.1_Candidatus_Nitrosocaldus_cavascurensis
3. Candidatus_Nitrosocaldus_schleperae_(JZ-1.bins.77)
4. WP_148681393.1_Candidatus_Nitrososphaera_gargensis_Ga9.2
5. WP_075055649.1_Nitrososphaera_viennensis_EN76
6. WP_144731398.1_Candidatus_Nitrosocosmicus_arcticus
7. WP_134482983.1_Candidatus_Nitrosocosmicus_franklandus
8. CUR52540.1_Candidatus_Nitrosotalea_devanaterra
9. WP_010194915.1_Candidatus_Nitrosarchaeum_limnium_BG20
10. WP_042683558.1_Candidatus_Nitrosotenuis_chungbukensis
11. WP_012215884.1_Nitrosopumilus_maritimus_SCM1
12. WP_048115106.1_Nitrosopumilus_adriaticus
B
AmoY
Identity
Sequence Logo
Transmembrane domain
1. CUR52554.1_Candidatus_Nitrosotalea_devanaterra
2. WP_012215889.1_Nitrosopumilus_maritimus_SCM1
3. WP_012215889.1_Nitrosopumilus_adriaticus
4. WP_007402986.1_Candidatus_Nitrosarchaeum_limnium_BG20
5. WP_042683693.1_Candidatus_Nitrosotenuis_chungbukensis
6. WP_048104617.1_Candidatus_Nitrosopelagicus_brevis
7. WP_015019253.1_Candidatus_Nitrososphaera_gargensis_Ga9.2
8. WP_227717441.1_Nitrososphaera_viennensis_EN76
9. WP_222424776.1_Candidatus_Nitrosocosmicus_arcticus
10. WP_197731110.1_Candidatus_Nitrosocosmicus_franklandus
11. WP_197706695.1_Candidatus_Nitrosocaldus_cavascurensis
12. Candidatus_Nitrosocaldus_schleperae_(JZ-1.bins.77)
13. Candidatus_Nitrosothermus_koennekei_(JZ-2.bins.172)
C
AmoZ
Identity
Sequence Logo
Transmembrane domain
1. WP_048115092.1_Nitrosopumilus_adriaticus
2. WP_012215890.1_Nitrosopumilus_maritimus_SCM1
3. WP_010194901.1_Candidatus_Nitrosarchaeum_limnium_BG20
4. WP_042683562.1_Candidatus_Nitrosotenuis_chungbukensis
5. WP_048106704.1_Candidatus_Nitrosopelagicus_brevis
6. CUR52555.1_Candidatus_Nitrosotalea_devanaterra
7. WP_144728940.1_Candidatus_Nitrosocosmicus_arcticus
8. WP_134483161.1_Candidatus_Nitrosocosmicus_franklandus
9. WP_148695127.1_Candidatus_Nitrosocaldus_cavascurensis
10. Candidatus_Nitrosocaldus_schleperae_(JZ-1.bins.77)
11. Candidatus_Nitrosothermus_koennekei_(JZ-2.bins.172)
12. WP_015019252.1_Candidatus_Nitrososphaera_gargensis_Ga9.2
13. WP_075053820.1_Nitrososphaera_viennensis_EN76

Figure S10. Alignments of archaeal AMO subunits from selected AOA species. A) AmoX, **B)** AmoY, and **C)** AmoZ. Predicted transmembrane helices based on the sequences from *N. viennensis* are indicated with an orange bars. In AmoZ **(C)**, cysteines predicted to form a disulfide bond between alpha helices in the soluble domain within the geneus *Nitrososphaera* and the family *Nitrosocaldaceae* are indicated with red boxes. The predicted zinc ribbon domain (cysteines indicated by black arrows) can be seen for the geneus *Nitrosocosmicus*.

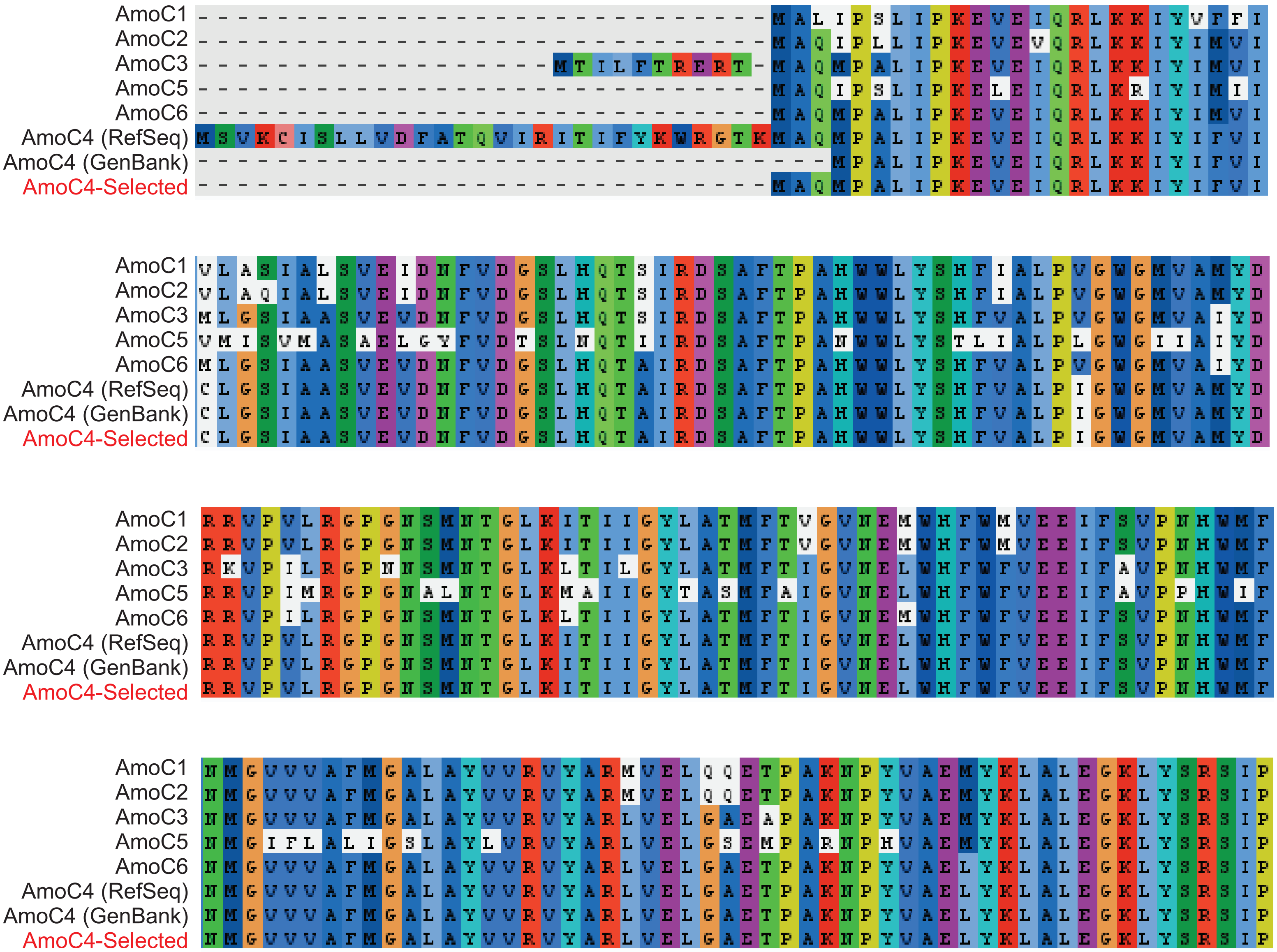
AmoC1
AmoC2
AmoC3
AmoC5
AmoC6
AmoC4 (RefSeq)
AmoC4 (GenBank)
AmoC4-Selected

Figure S11. AmoC homolog alignments from *N. viennensis*. Annotation differences can be seen for the AmoC4 homolog. The annotation labeled in red was chosen for analysis of AmoC peptides. Alignment created using AliView 1.27 [48].

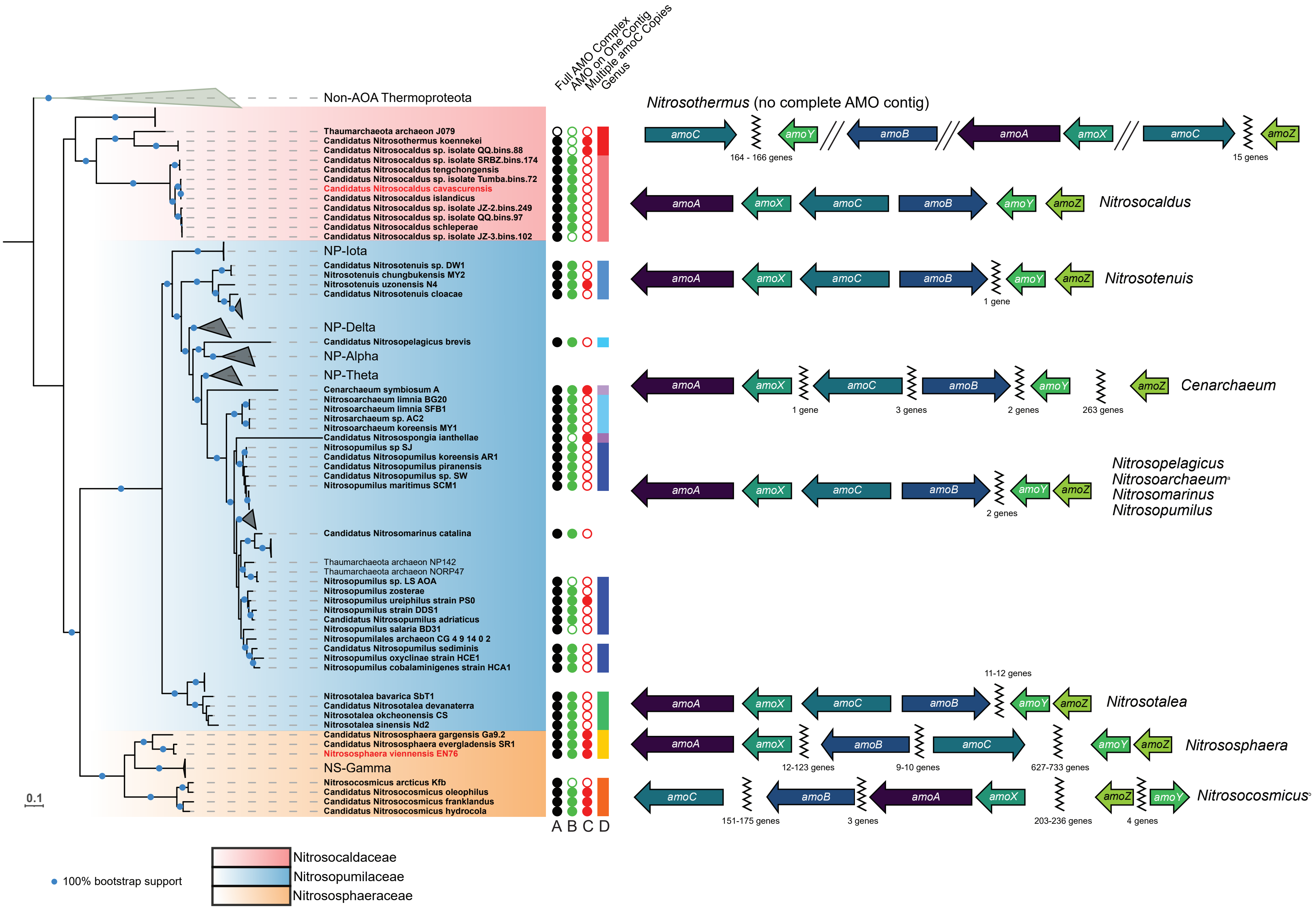
Full AMO Complex
AMO on One Contig
Multiple amoC Copies
Genus
Non-AOA Thermoproteota
Thaumarchaeota archaeon J079
Candidatus Nitrosothermus koennekei
Candidatus Nitrosocaldus sp. isolate QQ.bins.88
Candidatus Nitrosocaldus sp. isolate SRBZ.bins.174
Candidatus Nitrosocaldus tengchongensis
Candidatus Nitrosocaldus sp. isolate Tumba.bins.72
Candidatus Nitrosocaldus cavascurensis
Candidatus Nitrosocaldus islandicus
Candidatus Nitrosocaldus sp. isolate JZ-2.bins.249
Candidatus Nitrosocaldus sp. isolate QQ.bins.97
Candidatus Nitrosocaldus schleperae
Candidatus Nitrosocaldus sp. isolate JZ-3.bins.102
NP-Iota
Candidatus Nitrosotenuis sp. DW1
Nitrosotenuis chungbukensis MY2
Nitrosotenuis uzonensis N4
Candidatus Nitrosotenuis cloacae
NP-Delta
Candidatus Nitrosopelagicus brevis
NP-Alpha
NP-Theta
Cenarchaeum symbiosum A
Nitrosoarchaeum limnia BG20
Nitrosoarchaeum limnia SFB1
Nitrosarchaeum sp. AC2
Nitrosoarchaeum koreensis MY1
Candidatus Nitrosospongia ianthellae
Nitrosopumilus sp SJ
Candidatus Nitrosopumilus koreensis AR1
Candidatus Nitrosopumilus piranensis
Candidatus Nitrosopumilus sp. SW
Nitrosopumilus maritimus SCM1
Candidatus Nitrosomarinus catalina
Thaumarchaeota archaeon NP142
Thaumarchaeota archaeon NORP47
Nitrosopumilus sp. LS AOA
Nitrosopumilus zosterae
Nitrosopumilus ureiphilus strain PS0
Nitrosopumilus strain DDS1
Candidatus Nitrosopumilus adriaticus
Nitrosopumilus salaria BD31
Nitrosopumilales archaeon CG 4 9 14 0 2
Candidatus Nitrosopumilus sediminis
Nitrosopumilus oxyclinae strain HCE1
Nitrosopumilus cobalaminigenes strain HCA1
Nitrosotalea bavarica SbT1
Candidatus Nitrosotalea devanaterra
Nitrosotalea okcheonensis CS
Nitrosotalea sinensis Nd2
Candidatus Nitrososphaera gargensis Ga9.2
Candidatus Nitrososphaera evergladensis SR1
Nitrososphaera viennensis EN76
NS-Gamma
Nitrosocosmicus arcticus Kfb
Candidatus Nitrosocosmicus oleophilus
Candidatus Nitrosocosmicus franklandus
Candidatus Nitrosocosmicus hydrocola
A B C D
0.1
100% bootstrap support
Nitrosocaldaceae
Nitrosopumilaceae
Nitrososphaeraceae
Nitrosothermus (no complete AMO contig)
amoC amoY amoB amoA amoX amoC amoZ
164 - 166 genes
15 genes
amoA amoX amoC amoB amoY amoZ Nitrosocaldus
amoA amoX amoC amoB amoY amoZ Nitrosotenuis
1 gene
amoA amoX amoC amoB amoY amoZ Cenarchaeum
1 gene
3 genes
2 genes
263 genes
amoA amoX amoC amoB amoY amoZ
2 genes
Nitrosopelagicus
Nitrosoarchaeum
Nitrosomarinus
Nitrosopumilus
11-12 genes
amoA amoX amoC amoB amoY amoZ Nitrosotalea
amoA amoX amoB amoC amoY amoZ Nitrososphaera
12-123 genes
9-10 genes
627-733 genes
amoC amoB amoA amoX amoZ amoY Nitrosocosmicus
151-175 genes
3 genes
203-236 genes
4 genes

Figure S12. Genomic comparison of AMO subunit synteny in AOA (extended version of Figure 2). The left side of the figure represents a phylogenetic tree of AOA based on 32 conserved ribosomal proteins. Blue circles represent ultrafast bootstrap values of 100%. Taxonomic labels are colored according to GTDB family identity: *Nitrosocaldaceae*-red, *Nitrosopumilaceae*-blue, *Nitrososphaeraceae*-orange. Labels in bold were included in syntenic analysis. Labels in red represent species with proteomic evidence from BN-PAGE gels. Separate contigs are represented by a double forward slash. Gaps between genes on the same contig are marked by a zig-zag line. Numbers under the zig-zag lines represent number of genes between *amo* subunit genes. Added information next to the phylogenetic tree includes: (A) all six AMO subunits found (full circle); (B) AMO subunits found on the same contig (full circle); (C) multiple *amoC* copies found within the genome (full circle); (D) different genera indicated by different colors: "*Ca.* Nitrosothermus" (bright red), *Nitrosocaldus* (pink), *Nitrosotenuis* (sea blue), *Nitrosopelagicus* (aqua), "*Ca.* Cenarchaeum" (light purple), *Nitrosoarchaeum* (light blue), "*Ca.* Nitrosospongia*"* (dark purple), *Nitrosopumilus* (dark blue), *Nitrosotalea* (green), *Nitrosocosmicus* (yellow), *Nitrososphaera* (orange). With the exception of "*Ca.* Nitrosothermus sp.", species with AMO genes on different contigs could still plausibly fit into the shown arrangements. The sponge species "*Ca.* Nitrospongia ianthellae" and "*Ca*. Nitrosopumilus sp. LS AOA", as well as *Nitrosoarchaeum limnia* SFB1 differ from the other arrangements of closely related marine AOA by having one gene inserted between *amoYZ* and *amoABCX* rather than two.

Supplementary Material References

1. Schägger H, von Jagow G. Tricine-sodium dodecyl sulfate-polyacrylamide gel electrophoresis for the separation of proteins in the range from 1 to 100 kDa. *Anal Biochem* 1987; **166**: 368–379.

2. Schägger H. Tricine-SDS-PAGE. *Nat Protoc* 2006; **1**: 16–22.

3. Rath A, Deber CM. Correction factors for membrane protein molecular weight readouts on sodium dodecyl sulfate-polyacrylamide gel electrophoresis. *Anal Biochem* 2013; **434**: 67–72.

4. Altschul SF, Wootton JC, Gertz EM, Agarwala R, Morgulis A, Schäffer AA, et al. Protein database searches using compositionally adjusted substitution matrices. *FEBS J* 2005; **272**: 5101–5109.

5. Altschul SF, Madden TL, Schäffer AA, Zhang J, Zhang Z, Miller W, et al. Gapped BLAST and PSI-BLAST: a new generation of protein database search programs. *Nucleic Acids Res* 1997; **25**: 3389–3402.

6. Reyes C, Hodgskiss LH, Kerou M, Pribasnig T, Abby SS, Bayer B, et al. Genome wide transcriptomic analysis of the soil ammonia oxidizing archaeon *Nitrososphaera viennensis* upon exposure to copper limitation. *ISME J* 2020; **14**: 2659–2674.

7. Koo CW, Tucci FJ, He Y, Rosenzweig AC. Recovery of particulate methane monooxygenase structure and activity in a lipid bilayer. *Science* 2022; **375**: 1287–1291.

8. Lawton TJ, Ham J, Sun T, Rosenzweig AC. Structural conservation of the B subunit in the ammonia monooxygenase/particulate methane monooxygenase superfamily. *Proteins* 2014; **82**: 2263–2267.

9. Hyatt D, Chen G-L, LoCascio PF, Land ML, Larimer FW, Hauser LJ. Prodigal: prokaryotic gene recognition and translation initiation site identification. *BMC Bioinformatics* 2010; **11**: 119.

10. Katoh K, Misawa K, Kuma KI, Miyata T. MAFFT: A novel method for rapid multiple sequence alignment based on fast Fourier transform. *Nucleic Acids Res* 2002; **30**: 3059–3066.

11. Katoh K, Standley DM. MAFFT multiple sequence alignment software version 7: Improvements in performance and usability. *Mol Biol Evol* 2013; **30**: 772–780.

12. Camacho C, Coulouris G, Avagyan V, Ma N, Papadopoulos J, Bealer K, et al. BLAST+: Architecture and applications. *BMC Bioinformatics* 2009; **10**: 421.

13. Criscuolo A, Gribaldo S. BMGE (Block Mapping and Gathering with Entropy): A new software for selection of phylogenetic informative regions from multiple sequence alignments. *BMC Evol Biol* 2010; **10**: 210.

14. Minh BQ, Schmidt HA, Chernomor O, Schrempf D, Woodhams MD, Von Haeseler A, et al. IQ-TREE 2: New models and efficient methods for phylogenetic inference in the genomic era. *Mol Biol Evol* 2020; **37**: 1530–1534.

15. Hoang DT, Chernomor O, Von Haeseler A, Minh BQ, Vinh LS. UFBoot2: Improving the ultrafast bootstrap approximation. *Mol Biol Evol* 2018; **35**: 518–522.

16. Graham ED, Heidelberg JF, Tully BJ. Potential for primary productivity in a globally-distributed bacterial phototroph. *ISME J* 2018; **12**: 1861–1866.

17. Rinke C, Schwientek P, Sczyrba A, Ivanova NN, Anderson IJ, Cheng JF, et al. Insights into the phylogeny and coding potential of microbial dark matter. *Nature* 2013; **499**: 431–437.

18. Wittig I, Braun H-P, Schägger H. Blue native PAGE. *Nat Protoc* 2006; **1**: 418–428.

19. Reisinger V, Eichacker LA. Solubilization of membrane protein complexes for blue native PAGE. *J Proteomics* 2008; **71**: 277–283.

20. de Almeida NM, Wessels HJCT, de Graaf RM, Ferousi C, Jetten MSM, Keltjens JT, et al. Membrane-bound electron transport systems of an anammox bacterium: A complexome analysis. *Biochim Biophys Acta - Bioenerg* 2016; **1857**: 1694–1704.

21. Berger S, Cabrera-orefice A, Jetten MSM, Brandt U, Welte CU. Investigation of central energy metabolism-related protein complexes of ANME-2d methanotrophic archaea by complexome profiling. *BBA - Bioenerg* 2021; **1862**: 148308.

22. Hevler JF, Lukassen M V, Cabrera-Orefice A, Arnold S, Pronker MF, Franc V, et al. Selective cross-linking of coinciding protein assemblies by in-gel cross-linking mass spectrometry. *EMBO J* 2021; **40**: e106174.

23. Rappsilber J, Mann M, Ishihama Y. Protocol for micro-purification, enrichment, pre-fractionation and storage of peptides for proteomics using StageTips. *Nat Protoc* 2007; **2**: 1896–1906.

24. Tyanova S, Temu T, Cox J. The MaxQuant computational platform for mass spectrometry-based shotgun proteomics. *Nat Protoc* 2016; **11**: 2301–2319.

25. Perez-Riverol Y, Csordas A, Bai J, Bernal-Llinares M, Hewapathirana S, Kundu DJ, et al. The PRIDE database and related tools and resources in 2019: Improving support for quantification data. *Nucleic Acids Res* 2019; **47**: D442–D450.

26. Pirklbauer GJ, Stieger CE, Matzinger M, Winkler S, Mechtler K, Dorfer V. MS Annika: A new cross-linking search engine. *J Proteome Res* 2021; **20**: 2560–2569.

27. Iacobucci C, Götze M, Ihling CH, Piotrowski C, Arlt C, Schäfer M, et al. A cross-linking/mass spectrometry workflow based on MS-cleavable cross-linkers and the MeroX software for studying protein structures and protein–protein interactions. *Nat Protoc* 2018; **13**: 2864–2889.

28. Bullock JMA, Schwab J, Thalassinos K, Topf M. The importance of non-accessible crosslinks and solvent accessible surface distance in modeling proteins with restraints from crosslinking mass spectrometry. *Mol Cell Proteomics* 2016; **15**: 2491–2500.

29. Kong AT, Leprevost F V., Avtonomov DM, Mellacheruvu D, Nesvizhskii AI. MSFragger: Ultrafast and comprehensive peptide identification in mass spectrometry-based proteomics. *Nat Methods* 2017; **14**: 513–520.

30. Abby SS, Kerou M, Schleper C. Ancestral reconstructions decipher major adaptations of ammonia-oxidizing archaea upon radiation into moderate terrestrial and marine environments. *MBio* 2020; **11**: e02371-20.

31. Kim D, Paggi JM, Park C, Bennett C, Salzberg SL. Graph-based genome alignment and genotyping with HISAT2 and HISAT-genotype. *Nat Biotechnol* 2019; **37**: 907–915.

32. Liao Y, Smyth GK, Shi W. FeatureCounts: An efficient general purpose program for assigning sequence reads to genomic features. *Bioinformatics* 2014; **30**: 923–930.

33. Warnes GR, Bolker B, Bonebakker L, Gentleman R, Huber W, Liaw A, et al. gplots: Various R Programming Tools for Plotting Data. 2020. R package version 3.1.1.

34. R Core Team (2020). R: A language and environment for statistical computing. 2020. R Foundation for Statistical Computing, Vienna, Austria.

35. Eren AM, Kiefl E, Shaiber A, Veseli I, Miller SE, Schechter MS, et al. Community-led, integrated, reproducible multi-omics with anvi'o. *Nat Microbiol* 2021; **6**: 3–6.

36. Capella-Gutiérrez S, Silla-Martínez JM, Gabaldón T. trimAl: A tool for automated alignment trimming in large-scale phylogenetic analyses. *Bioinformatics* 2009; **25**: 1972–1973.

37. Käll L, Krogh A, Sonnhammer ELL. A combined transmembrane topology and signal peptide prediction method. *J Mol Biol* 2004; **338**: 1027–1036.

38. Jumper J, Evans R, Pritzel A, Green T, Figurnov M, Ronneberger O, et al. Highly accurate protein structure prediction with AlphaFold. *Nature* 2021; **596**: 583–589.

39. Varadi M, Anyango S, Deshpande M, Nair S, Natassia C, Yordanova G, et al. AlphaFold Protein Structure Database : massively expanding the structural coverage of protein-sequence space with high-accuracy models. *Nucleic Acids Res* 2022; **50**: D439–D444.

40. Evans R, O'Neill M, Pritzel A, Antropova N, Senior A, Green T, et al. Protein complex prediction with AlphaFold-Multimer. *bioRxiv* 2022; doi: 10.1101/2021.10.04.463034.

41. Almagro Armenteros JJ, Tsirigos KD, Sønderby CK, Petersen TN, Winther O, Brunak S, et al. SignalP 5.0 improves signal peptide predictions using deep neural networks. *Nat Biotechnol* 2019; **37**: 420–423.

42. Schrodinger LLC. The PyMOL Molecular Graphics System, Version 1.8. 2015.

43. Luo Z-H, Narsing Rao MP, Chen H, Hua Z-S, Li Q, Hedlund BP, et al. Genomic insights of "*Candidatus* Nitrosocaldaceae" based on nine new metagenome-assembled genomes, including "*Candidatus* Nitrosothermus" gen nov. and two new species of "*Candidatus* Nitrosocaldus". *Front Microbiol* 2021; **11**: 608832.

44. Hakemian AS, Kondapalli KC, Telser J, Hoffman BM, Stemmler TL, Rosenzweig AC. The metal centres of particulate methane monooxygenase from *Methylosinus trichosporium* OB3b. *Biochemistry* 2008; **47**: 6793–6801.

45. Lieberman RL, Rosenzweig AC. Crystal structure of a membrane-bound metalloenzyme that catalyses the biological oxidation of methane. *Nature* 2005; **434**: 177–182.

46. Berman HM, Westbrook J, Feng Z, Gary G, Bhat TN, Weissig H, et al. The protein data bank. *Nucleic Acids Res* 2000; **28**: 235–242.

47. Rinke C, Chuvochina M, Mussig AJ, Chaumeil PA, Davín AA, Waite DW, et al. A standardized archaeal taxonomy for the Genome Taxonomy Database. *Nat Microbiol* 2021; **6**: 946–959.

48. Larsson A. AliView: A fast and lightweight alignment viewer and editor for large datasets. *Bioinformatics* 2014; **30**: 3276–3278.
